# Quantifying neutrophil extracellular trap release in a combined infection–inflammation NET-array device†

**DOI:** 10.1039/d3lc00648d

**Published:** 2024-01-04

**Authors:** Udaya Sree Datla, Bhaskar Vundurthy, Jessica S. Hook, Nidhi Menon, Hossein Razmi Bagtash, Tarik Shihabeddin, David W. Schmidtke, Jessica G. Moreland, Marko Z. Radic, Caroline N. Jones

**Affiliations:** a Translational Biology, Medicine and Health, Virginia Polytechnic Institute and State University Blacksburg VA USA; b Department of Bioengineering, University of Texas at Dallas Richardson TX USA Caroline.jones@utdallas.edu; c Department of Surgery, University of Texas Southwestern Medical Center Dallas TX USA; d Robotics Institute, Carnegie Mellon University Pittsburgh PA USA; e Department of Pediatrics, University of Texas Southwestern Medical Center Dallas TX USA; f Department of Microbiology, University of Texas Southwestern Medical Center Dallas TX USA; g Department of Microbiology, Immunology and Biochemistry, University of Tennessee Health Science Center Memphis TN USA

## Abstract

Excessive release of neutrophil extracellular traps (NETs) has been reported in various human pathologies, including COVID-19 patients. Elevated NET levels serve as a biomarker, indicating increased coagulopathy and immunothrombosis risks in these patients. Traditional immunoassays employed to quantify NET release focus on bulk measurements of released chromatin in simplified microenvironments. In this study, we fabricated a novel NET-array device to quantify NET release from primary human neutrophils with single-cell resolution in the presence of the motile bacteria *Pseudomonas aeruginosa* PAO1 and inflammatory mediators. The device was engineered to have wide chambers and constricted loops to measure NET release in variably confined spaces. Our open NET-array device enabled immunofluorescent labeling of citrullinated histone H3, a NET release marker. We took time-lapse images of primary healthy human neutrophils releasing NETs in clinically relevant infection and inflammation-rich microenvironments. We then developed a computer-vision-based image processing method to automate the quantification of individual NETs. We showed a significant increase in NET release to *Pseudomonas aeruginosa* PAO1 when challenged with inflammatory mediators tumor necrosis factor-α [20 ng mL^−1^] and interleukin-6 [50 ng mL^−1^], but not leukotriene B_4_ [20 nM], compared to the infection alone. We also quantified the temporal dynamics of NET release and differences in the relative areas of NETs, showing a high percentage of variable size NET release with combined PAO1 – inflammatory mediator treatment, in the device chambers. Importantly, we demonstrated reduced NET release in the confined loops of our combined infection–inflammation microsystem. Ultimately, our NET-array device stands as a valuable tool, facilitating experiments that enhance our comprehension of the spatiotemporal dynamics of NET release in response to infection within a defined microenvironment. In the future, our system can be used for high throughput and cost-effective screening of novel immunotherapies on human neutrophils in view of the importance of fine-tuning NET release in controlling pathological neutrophil-driven inflammation.

## Abstract 2

We present a neutrophil extracellular trap (NET)-array device, engineered to quantify the spatiotemporal dynamics of NET release with single-cell resolution in varied microenvironments simulating infection and inflammation.

## Graphical abstract

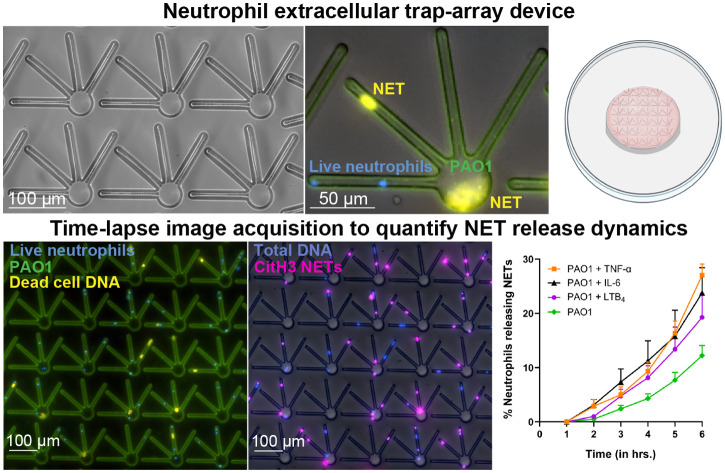

## Introduction

Neutrophils, the most abundant immune cell type, and early responders to infection, are effectively guided to infection sites through chemotactic signals from invaders and damaged host tissue. Upon reaching the site of infection, neutrophils deploy defense mechanisms to trap or neutralize the pathogen *via* phagocytosis, swarming, degranulation, oxidative burst, and by releasing DNA as neutrophil extracellular traps (NETs).^1–13^ While NETs primarily aim to defend the host against infection, aberrant NET release or inadequate clearance of NETs may lead to host tissue damage,^14,15^ besides contributing to blood clotting and formation of thrombi.^16^ Research indicates that NETs aggregate and occlude the ducts of various organs like the pancreas and gall bladder.^17,18^ Additionally, they obstruct the microvasculature in the lungs, liver, kidneys, and heart, contributing to the development of disease.^19–21^ NETs contain a range of antimicrobial peptides, histones and proteases, which can have deleterious effects. Excessive NET release can adversely affect individuals with immunopathological conditions related to cystic fibrosis,^15^ autoimmune diseases,^22–24^ cardiovascular complications,^25^ cancer,^26^ sepsis^27^ and COVID-19.^28,29^ The cytokine storm driving the overzealous host immune responses manifests in exacerbated levels of tissue inflammation and ultimately organ failure, in severe cases of sepsis and SARS-CoV-2 infections.^15,27–30^ Of the inflammatory mediators, leukotriene B_4_ (LTB_4_), tumor necrosis factor-α (TNF-α) and interleukin-6 (IL-6) are highly upregulated in patients suffering from inflammatory conditions with dysregulated NET release, namely sepsis and COVID-19.^31–36^ The accumulation of NETs in the lungs of severe COVID-19 patients can exacerbate lung damage and contribute to the development of acute respiratory distress syndrome (ARDS), a critical complication of the disease.^28,29^ Therefore, it is essential to take the inflammatory mediator-rich microenvironment into account, while quantifying neutrophil interactions with the pathogen during an infection, focusing on NET release dynamics.

Individuals with compromised immune responses are especially vulnerable to life-threatening secondary bacterial infections from *Pseudomonas aeruginosa* – one of the leading causes (18–20%) of hospital-acquired lung infections with high antibiotic resistance and present huge clinical challenges.^37^*P. aeruginosa* infection is also lethal to premature babies and the elderly in ICUs who require the use of invasive devices like IV catheters and breathing tubes (the major sources of infections). Notably, *P. aeruginosa* can establish persistent lung infections in patients with respiratory diseases like cystic fibrosis (CF), with neutrophils releasing abundant NETs and driving inflammation in CF airways.^37–39^ Prior studies showed that both the laboratory strains and the CF clinical isolates of *P. aeruginosa* strongly induced NET release *in vitro*.^40–43^*P. aeruginosa* in planktonic, flagellated forms induced higher NET release than flagellum-deficient bacteria, showing that flagellar motility is an essential mediator of NET release.^40^ Despite extensive research on NETs and bacteria interaction, the precise nature of how microenvironmental signals influence NET release while mediating inflammation during bacterial infections is yet to be fully understood. While the traditional *in vitro* assays are often diverse and bulk assays that do not reflect on the individual neutrophil heterogeneity and polarization states,^44^ the lack of control of the complex tissue microenvironment *in vivo* limits our understanding of the NET release dynamics.

To address this, inflammation-on-chip technologies have been developed to enable precise measurements of NET release in a well-defined microenvironment. Microtechnologies developed to study the NET release dynamics include devices for simultaneous quantification of NETs and reactive oxygen species (ROS) production to *P. aeruginosa* infection,^45^ and the ones that were used to measure cell-free DNA concentration originating from circulating NETs (cNETs) in blood showing increased intact cNETs after burn injury and secondary sepsis in rats.^46^ More recently, a novel flow-regulated NET-capturing microfluidic system was developed to measure NET release *via* trapping chromatin fibers from human whole blood,^47^ which was later used to study the resolution function of T-series resolvins in reducing NETs.^34^ While these microdevices with built-in microscale architecture (channels and valves) offer the added advantage of a reproducible and controlled microenvironment to quantify the NET release dynamics at a high resolution,^48–50^ their closed microarchitecture makes the post-processing of NETs (such as immunofluorescent labeling of specific NET proteins) challenging.

**Fig. 1:**
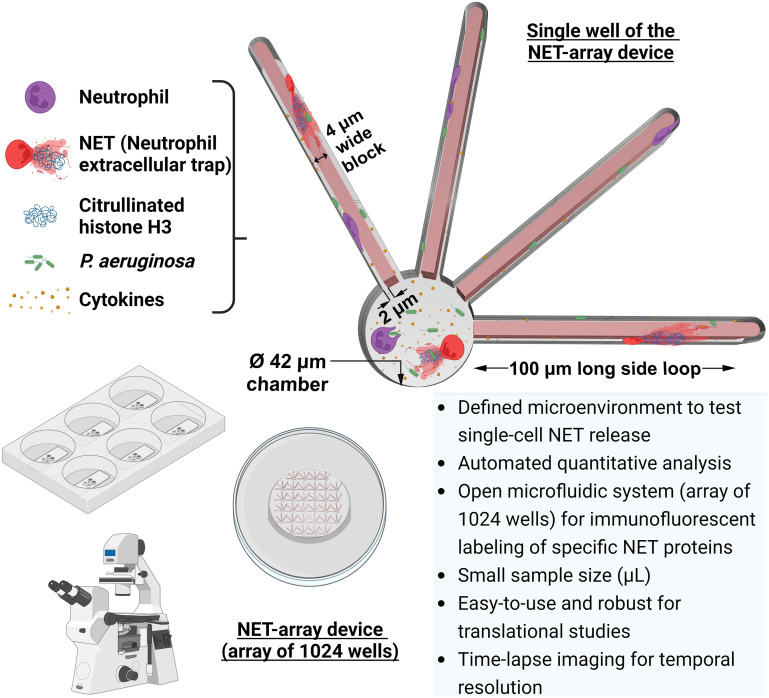
Overview of the combined infection–inflammation NET-array device to quantify the dynamic neutrophil extracellular trap (NET) release. Our custom NET-array device is an open microfluidic platform that stations 1024 wells per device and is used to investigate the spatiotemporal dynamics of single-cell NET release in a precisely defined microenvironment. The device is integrated with an automated quantification system that enables us to quantify NET release through a live-dead cell assay, followed by immunofluorescent labeling of specific NET proteins (citrullinated histone H3).

To further characterize the spatiotemporal dynamics of NET release during infection, we engineered and integrated a novel NET-array device with a time-lapse imaging assay to quantify the NETs released in a regulated microenvironment at a single-cell resolution. In our study, we explored how the motile strain of *P. aeruginosa* PAO1-GFP, when planktonic, affects NET release dynamics differently in the presence of inflammatory mediators like leukotriene B_4_, tumor necrosis factor-α, and interleukin-6. We concentrated on the spatial (by measuring decondensed chromatin areas in neutrophils) and temporal factors of NET release within a microsystem that combines infection and inflammation. We developed a computational pipeline to automatically classify and categorize NETs based on chromatin areas, further tracking the precise location of NET release in the individual wells of the device. Besides offering some of the benefits of other microfluidic devices, our NET-array device features also enable us to study NET release in variably confined spaces. The relatively confined side loops in our device mimic the narrow capillary segments in the pulmonary and systemic microcirculation that neutrophils encounter while migrating *in vivo*. Following neutrophil trapping, we confined motile *P. aeruginosa* bacteria in individual wells, using special covers to seal the device, and better understand neutrophil–*P. aeruginosa* interactions (Fig. 1). We showed that the presence of the inflammatory mediators TNF-α and IL-6 in conjunction with *P. aeruginosa* triggered a higher percentage of the primary healthy human neutrophils to release NETs, compared to *P. aeruginosa* with no inflammatory mediators, in our NET-array device. Further, we ran a comparative analysis and demonstrated lower NET release from neutrophils confined in the relatively narrow side loops (2 μm width) compared to the chambers (42 μm diameter) in our combined infection–inflammation microsystem. Unlike the other *in vitro* assays, we reported no aggregation/entanglement of NETs released from multiple neutrophils in our device, making single-cell tracking and automated quantification easier. Importantly, our open well device design allows reproducible immunofluorescence results to stain the markers of NET release (citrullinated histone H3), unlike the closed microdevices that use a tedious process of using pumps to flow the reagents through the device to stain NETs. During NET formation, neutrophils undergo chromatin decondensation, promoted by citrullination of histone H3, a critical event that results in the relaxation of chromatin and its subsequent release into the extracellular space as NETs.^51–53^ The presence of citrullinated histone H3 serves as a specific and reliable marker for the formation of NETs. Citrullinated histone H3 has also been used as a clinical biomarker for cancer and the severity of other inflammatory diseases.^54–56^ In summary, our results highlight the role of the mechanochemical complexity of the microenvironment on the release of NETs in host defense and inflammation.

## Materials and methods

### Microfluidic device design and fabrication

AutoCAD (Autodesk, San Rafael, CA) was used to design the device, and Front Range Photomask (Lake Havasu City, AZ) to develop the chrome mask. The NET-array devices used to quantify the NET release dynamics at a single-cell level were fabricated using standard microfabrication techniques. First, SU-8 25 photoresist (Kayaku Advanced Materials, Westborough, MA) was spin-coated on the silicon wafer at 38 μm thickness. The device features were then patterned on the coated photoresist using the photolithographic mask and processed according to the manufacturer's instructions. This patterned wafer was then silanized with trichlorosilane(trichloro(1*H*,1*H*,2*H*,2*H*-perfluorooctyl)silane, Sigma-Aldrich, St. Louis, MO) in a vacuum chamber and used as a master mold to produce polydimethylsiloxane (PDMS) devices. The PDMS devices were prepared by thoroughly mixing the monomer base and curing agents (Dow Sylgard 184 silicone elastomer kit, Ellsworth Adhesives, Germantown, WI) at a 10 : 1 ratio and degassing under vacuum for about 1 h. This was followed by spin coating the mix on the silicon master wafer to produce a 0.6 to 0.7 mm thick PDMS mold, which was then cured at 80 °C for 1 h. Later, the PDMS devices were cut, plasma bonded in a glass-bottom well plate with the NET-array wells facing up, and ready to use.

### Isolation of primary human neutrophils from healthy volunteers

To quantify the differences in the NET release dynamics in fighting the planktonic infections of *P. aeruginosa*, we collected whole blood samples in ACD tubes (acid citrate dextrose tubes, Becton Dickinson, Franklin Lakes, NJ) from individual healthy volunteers at the University of Texas Southwestern Medical Center. Written informed consent was obtained from the healthy volunteers to draw blood for the study and to publish non-identifiable data. This was done in accordance with the study number STU 012014-040, approved by the Institutional Review Board at the University of Texas Southwestern Medical Center. We isolated the neutrophils from these samples using the EasySep™ direct human neutrophil isolation kit (STEMCELL Technologies, Cambridge, MA), which employs the immunomagnetic negative selection principle to isolate neutrophils directly from human whole blood. Prior to use in the assay, the neutrophils were pre-stained at 10^6^ neutrophils per mL in 32.4 μM of Hoechst 33342 solution (10 mg mL^−1^ stock solution in water, Invitrogen, Waltham, MA) for 20 min in an incubator at 37 °C, followed by 2× washing in the serum-free buffer to remove extra stain from the solution. Stained neutrophils were then suspended in the assay buffer at a concentration of 1.5–2 × 10^5^ cells per mL. Assay buffer: NETs assay buffer was prepared from hanks balanced salt solution (HBSS, 1× with calcium and magnesium, Corning, NY) by adding 5 mM glucose (200 g L^−1^ stock solution, Gibco, Waltham, MA), 10 mM HEPES (1 M stock solution, Gibco, Waltham, MA) and 1% autologous serum (collected from the donor), mixed well. Serum-free buffer was prepared the same way as the assay buffer excluding the addition of serum.

### *Pseudomonas aeruginosa* culture

*Pseudomonas aeruginosa* (the standard lab strain PAO1) culture was grown overnight in TSB (tryptic soy broth, Becton Dickinson, Franklin Lakes, NJ) supplemented with 200 μg mL^−1^ carbenicillin (Sigma-Aldrich, St. Louis, MO), in a shaking incubator at 37 °C. The next day, they were subcultured at 1/100 dilution in TSB and incubated at 37 °C for about 5 h until the OD_600nm_ reached 0.6 (where the cell density is approximately 2 × 10^8^ CFU mL^−1^). These cultures were then pelleted at 2500×*g* for 10 min at 4 °C, the supernatant was discarded, and then the pellets were washed 1× with serum-free buffer. After 1× wash, the pellets were suspended to the original volume with the assay buffer and placed on ice. Before use in the assay, the cell suspension was diluted 1/100 in the assay buffer to adjust an MOI (multiplicity of infection) of 10 with neutrophils. The *P. aeruginosa* PAO1 strain used has a constitutive *gfp* expressing plasmid PMRP9-1 (selected using carbenicillin resistant marker) for the production of GFP for use in live imaging.^57^

### Microfluidic assay to quantify the NET release dynamics in fighting *P. aeruginosa* planktonic infection in the NET-array device

The devices bonded in the glass bottom well plate were initially plasma treated to render their surface hydrophilic, followed by adding 50 μL of PBS. The setup was then placed in a vacuum desiccator for about 1.5 h to prime the devices and remove the air trapped in the devices. After that, PBS was removed and 40 μL of 11 μg mL^−1^ human fibronectin (0.1% stock solution, Sigma-Aldrich, St. Louis, MO) was added on top of each device and left to air dry for 20 min. The extra fibronectin was removed by aspiration, followed by 1× wash with the assay buffer. Fibronectin was added to aid the adhesion of the neutrophils while releasing NETs or when moving inside the device. Following this, 40 μL of the pre-stained neutrophil cell suspension was added to the device and allowed the neutrophils to settle in the device wells for 5 to 10 min. Extra buffer and cells outside the wells were removed by aspiration, followed by 1× wash with the assay buffer. Finally, 40 μL of the diluted *P. aeruginosa* (PAO1) cell suspension mixed with 5 μM Sytox orange (5 mM stock solution in DMSO, Invitrogen, Waltham, MA) was added to the devices. The inflammatory mediators LTB_4_/TNF-α/IL-6 (LTB_4_ from Cayman Chemical Company, Ann Arbor, MI and TNF-α, IL-6 – animal-free recombinant human cytokines from PeproTech, Cranbury, NJ) were added to this final solution at the test concentration. Phorbol 12-myristate 13-acetate (PMA) (Sigma-Aldrich, St. Louis, MO) is a NET stimulant added as positive control where relevant. The assay buffer and *P. aeruginosa* were then allowed to settle in the device wells for 10 min, and 20 μL of the buffer was removed from the top of each device to prevent spillage of buffer under the press-to-seal cover in the next step. Finally, the devices were sealed with 0.6 mm thick press-to-seal covers, and serum-free buffer was added outside on top of the covers to prevent evaporation of buffer from the devices. The selected neutrophil behavior (NET release) was then imaged in real-time using a time-lapse Nikon Ti2 microscope (in 20× magnification) at 37 °C for a total of 6 h (with 1 h time interval). After the live imaging, we removed the covers gently and proceeded to immunofluorescent label the neutrophils on-chip to stain markers of NET release. The protocol is outlined in the schematic in Fig. S1.†

### Immunocytochemistry on-chip to study NET release

After the NETs assay, the press-to-seal cover was gently lifted up, and the assay buffer was aspirated from the device wells. Then, the cells were fixed with 4% PFA (paraformaldehyde, 16%, methanol free, Polysciences, Warrington, PA) for 15 min at room temperature. Following the fixation, the cells were washed 3× with PBS at 5 min/wash. Later, the cells were blocked with 10% normal goat serum (Cell Signaling Technology, Danvers, MA) for 1 h at room temperature. The blocking buffer was then removed, and the cells were incubated in 4 μg mL^−1^ of the primary antibody (rabbit polyclonal anti-histone H3 (citrulline R2 + R8+ R17) antibody, Abcam, Waltham, MA) in 1% BSA + 1% goat serum in PBS buffer for 2 h at room temperature. The cells were then washed 3× with PBS at 5 min/wash. Finally, the cells were incubated in 20 μg mL^−1^ of the secondary antibody (goat anti-rabbit IgG H&L Alexa Fluor 647 pre-adsorbed, Abcam, Waltham, MA) in 1% BSA + 1% goat serum in PBS buffer for 1 h at room temperature (in the dark). The cells were then counter-stained with Hoechst solution (10 μg mL^−1^), followed by 2× washing with PBS at 5 min/wash and imaged.

### Automated image analysis

Our custom NET-array image analysis addressed the unique challenge of determining if a detected cell lies inside or outside the boundary of individual wells in the device. We leveraged the Image Processing Toolbox from MATLAB to create a custom script (https://github.com/bvundurthy/NET-array) that first determines the locations of all the wells in the device, followed by the detection of live and dead cells across all time points (Fig. S2A†). The script applied a Laplacian of Gaussian (LoG) filter to the bright field image to create a high contrast near the well boundary. The wells are then identified by computing the zero-crossing in the filtered image (Fig. S2B†). To distinguish between the circular chambers and side loops of individual wells, the script employed circular Hough transform to detect the chambers (marked in green in Fig. S2B†) and classified the remainder of the wells as loops (marked in white in Fig. S2B†). The cells (Fig. S2C†), on the other hand, have irregular boundaries and thus mandate morphological operations on the DAPI image prior to the application of the LoG filter and computation of zero-crossings. Any small displacements in the well location are handled by using the *k*-nearest neighbors' algorithm to associate cells and wells with their prior counterparts. The script outputs the area (Fig. S2C†), centroid, and bounding box information for live cells that lie within either loops or chambers, followed by characterizing NET release for individual cells. To quantify NETs at a single-cell resolution using our automated analysis pipeline, we considered only wells with 1–4 neutrophils deposited in them to exclude aggregated NETs (resulting from a higher neutrophil deposition density) from the analysis. We then randomly selected 100 neutrophils from chambers and 100 from loops and classified only those neutrophils with an increase in chromatin areas of the dead neutrophils at least double the area of their live nuclei, as releasing NETs. We repeated the randomization process (ensuring no overlap in neutrophils analyzed) and reported the average values per volunteer.

### Statistical analysis

All the experimental conditions were tested using primary neutrophils from 5 to 9 individual healthy human donors unless stated otherwise. Data were expressed as mean ± standard error of mean and considered statistically significant where *p* ≤ 0.05. Graphs were plotted, and statistical analyses were performed using GraphPad software (La Jolla, CA). Two-tailed student *t*-test was used to compare the differences in NET release between two treatment groups or treatment *versus* control, whereas a paired student *t*-test was used to compare NET release between loops *versus* chambers of individual treatment groups. While one-way or two-way ANOVA with *post hoc* tests were used for multiple group comparisons.

## Results

### NET-array device design and assay to study the dynamics of NET release in a combined infection–inflammation microarray system

**Fig. 2:**
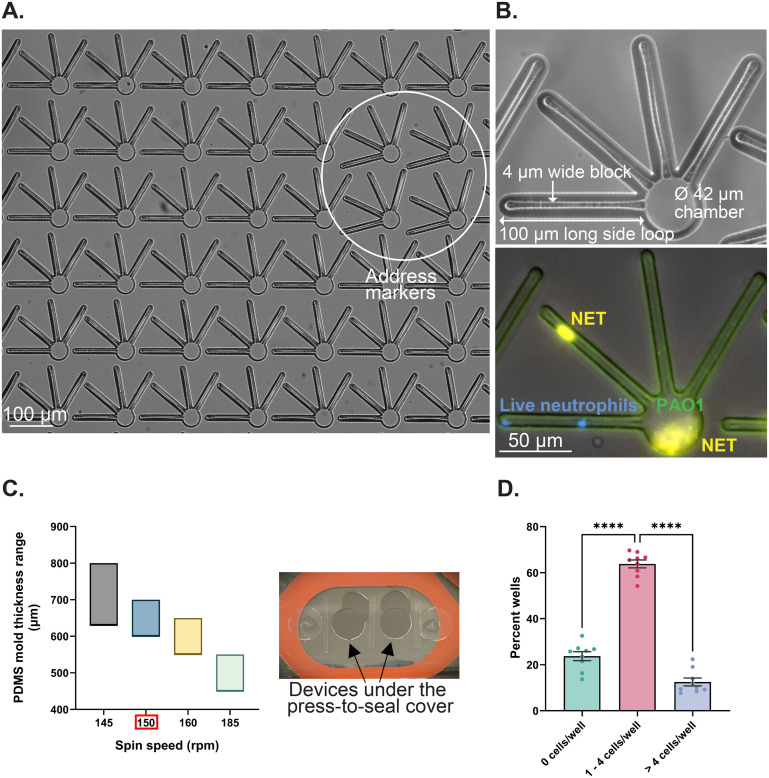
NET-array device design and optimization to study the spatiotemporal dynamics of NET release. (A) Brightfield image showing 6 × 7 wells of the NET-array device (wells that are rotated by 15° serve as address markers and help in streamlining the image acquisition). (B) Top: Single well of the NET-array device showing a magnified view of the circular chamber and four side loops. Each side loop measures 100 μm long × 2 μm wide and runs around a 4 μm wide block. Bottom: Fluorescent image of a single well showing NET release to PAO1 in the side loops *versus* the chamber of the device (blue: live neutrophil DNA (Hoechst 33342 stained); orange: NETs (Sytox orange stained); green: PAO1-GFP). (C) Left: Range of the thickness of the PDMS mold (μm) spread on the silicon master wafer as a function of the spin-coating speed (rpm). Right: Press-to-seal cover to seal the devices thereby confining the neutrophils and *P. aeruginosa* in the device wells and assisting the live imaging of their interactions. (D) Distribution of neutrophil deposition density in individual wells of the NET-array device (neutrophils seeded at a cell density of 1.5–2 × 10^5^ cells per mL and volume of neutrophil suspension at 40 μL). ****p < 0.0001; one-way ANOVA with Tukey's *post hoc* test used. Data are represented as mean ± SEM. *N* = 39 different devices and neutrophils isolated from 9 different donors.

To quantify the spatiotemporal dynamics of NET release to *Pseudomonas aeruginosa* infection in a controlled microenvironment, we developed NET-array devices with 1024 wells (32 × 32 array) in each device. Wells are rotated by 15° at specific locations in the microarray to serve as address markers (Fig. 2A) for better image streamlining and easy retrieval of the neutrophils of interest for future single-cell analyses. Individual wells are 38 μm deep and designed to have a 42 μm wide circular core region (referred to as chambers), spanning four side loops with each measuring 100 μm long × 2 μm wide, around a 4 μm wide block (Fig. 1 and 2B). We achieved deposition of neutrophils and *P. aeruginosa* in both the chambers and loops (Fig. 2B). The narrow side loops in our microsystem enabled us to study NET release behavior in relatively confined spaces, owing to the transit of neutrophils through disseminated infection in microvasculature and mechanically complex tissue spaces. The device design was optimized to enable the precise quantification of NET release dynamics, measuring the areas and time of NETs released in chambers *versus* side loops of the device during a *P. aeruginosa* PAO1 infection in a controlled microenvironment. To monitor the confinement of neutrophils and *P. aeruginosa* in the device, we adjusted the spin coating speed of PDMS at 150 rpm to generate 0.6 to 0.7 mm thick devices that fit under the 0.6 mm thick press-to-seal cover (Fig. 2C). The press-to-seal cover aids in sealing the device for time-lapse imaging. When 40 μL of the neutrophil cell suspension with a seeding density of 1.5–2 × 10^5^ cells per mL was added to the device, the volume was enough to prime all the wells in the device, and >60% of the wells had 1–4 neutrophils each (Fig. 2D). The details of the assay quantifying the NET release dynamics when encountered with the *P. aeruginosa* planktonic infection under different treatments, are listed in the methods section.

### Spatiotemporal dynamics of NET release to *P. aeruginosa* PAO1 infection in inflammatory mediator-rich microenvironment

**Fig. 3:**
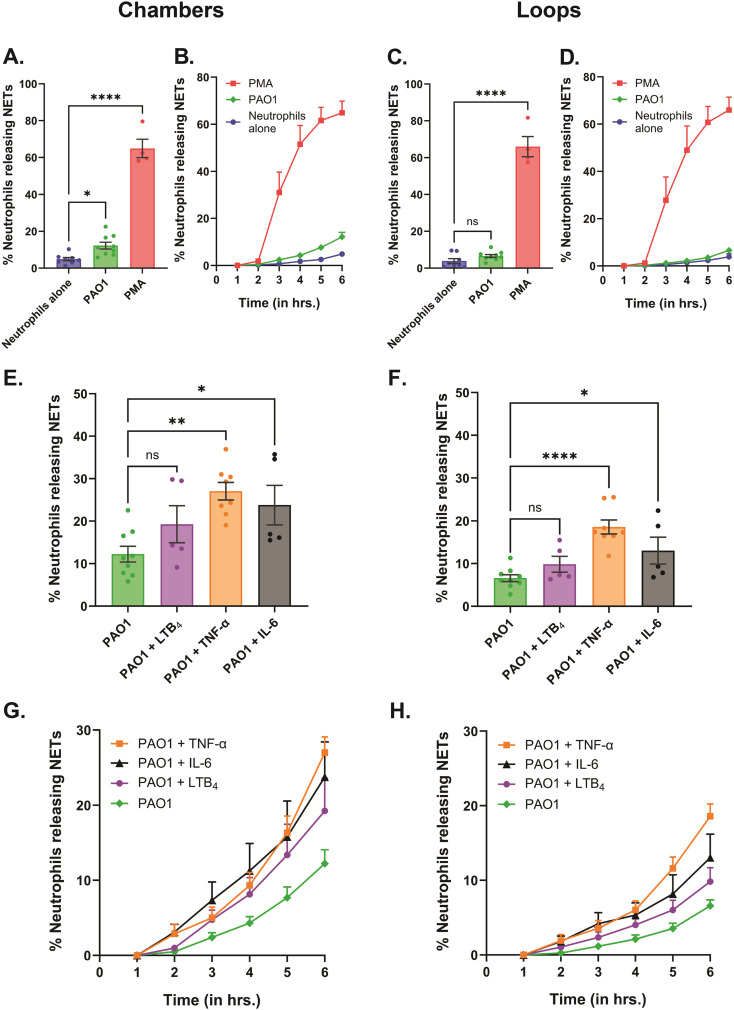
Dynamics of the NETs assay recorded for 6 h, showing increased NET release to *P. aeruginosa* (PAO1) when treated with the inflammatory mediators TNF-α and IL-6. Data are represented as mean ± SEM and collected from 5 to 9 separate healthy volunteers, except for positive control where *n* = 4 donors. 20 nM PMA was used as the positive control to stimulate NET release, while neutrophils (without PAO1 and inflammatory mediators) were used as the negative control in the assay. Percentage of neutrophils releasing NETs over 6 h with PMA [20 nM] or PAO1 compared to the negative control, in the (A) chambers and (C) loops of the device. ns – not significant, *p < 0.05, ****p < 0.0001; one-way ANOVA with Dunnett's multiple comparisons test used to compare NET release between treatment groups and negative control. Temporal dynamics of percent NET release with PMA [20 nM] or PAO1 compared to the negative control, in the (B) chambers and (D) loops of the device. Graphs comparing the percentage of neutrophils releasing NETs over 6 h to PAO1 with or without the inflammatory mediators 20 nM LTB_4_, 20 ng mL^−1^ TNF-α and 50 ng mL^−1^ IL-6 respectively, in the (E) chambers and (F) loops of the device. ns – not significant, *p < 0.05, **p < 0.01, ****p < 0.0001; one-way ANOVA with Dunnett's multiple comparisons test used to compare NET release between treatment groups and PAO1 control. Temporal dynamics of percent NET release to PAO1 with or without the inflammatory mediators 20 nM LTB_4_, 20 ng mL^−1^ TNF-α and 50 ng mL^−1^ IL-6, respectively, in the (G) chambers and (H) loops of the device.

**Fig. 4:**
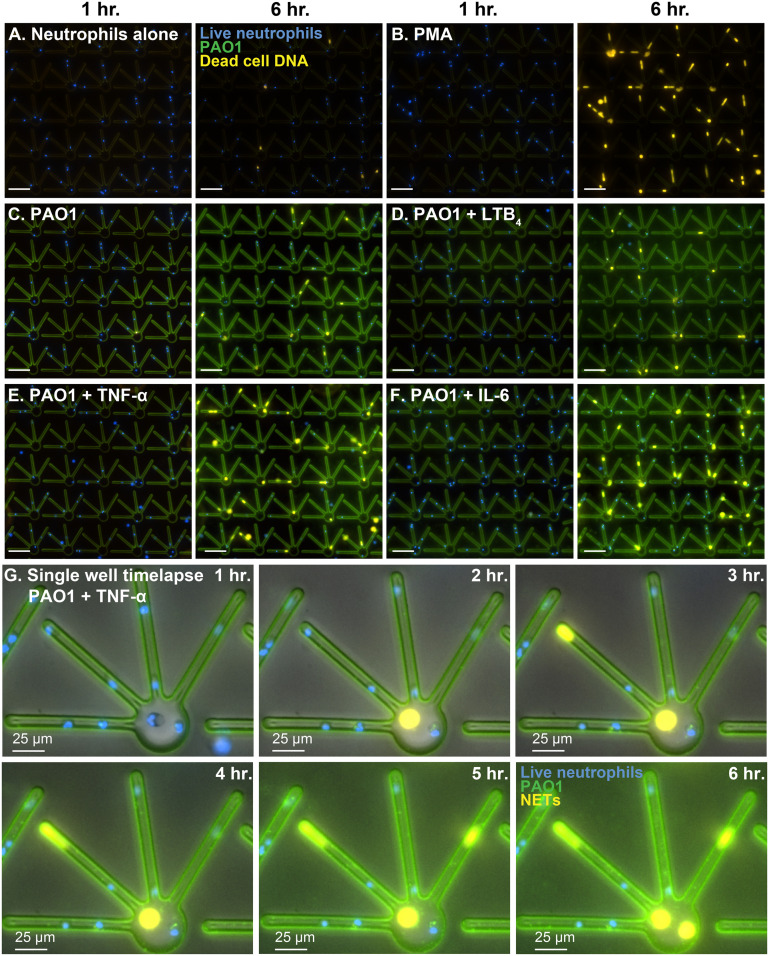
NET release to *P. aeruginosa* (PAO1) in the device, at 1 h and 6 h respectively, with multiple treatment groups. Refer to Video S1† for the time-lapse movie. Blue: live neutrophil DNA (Hoechst 33342 stained); orange: dead cell DNA (Sytox orange stained); green: PAO1-GFP. (A) Negative control (neutrophils alone) (B) positive control (20 nM PMA) (C) neutrophils + PAO1 (no inflammatory mediator) (D) neutrophils + PAO1 + 20 nM LTB_4_ (E) neutrophils + PAO1 + 20 ng mL^−1^ TNF-α and (F) neutrophils + PAO1 + 50 ng mL^−1^ IL-6. Scale bar: 100 μm. (G) Detailed images of a single well from a 6 h long timelapse setup, showing NET release dynamics to PAO1 + 20 ng mL^−1^ TNF-α in the 100 μm long side loops *versus* the chambers of the device.

To investigate the differences in NET release behavior in primary healthy human neutrophils when encountered with *P. aeruginosa* (PAO1) infection in an inflammation-rich microenvironment, we ran the live-imaging assay. Neutrophils were deposited in the wells of individual devices, followed by the addition of PAO1 (MOI 10), with or without the inflammatory mediators (LTB_4_/TNF-α/IL-6) for the imaging experiment. After the bacteria had been deposited in the wells, we sealed the devices with 0.6 mm thick custom press-to-seal covers and set up the timelapse imaging. As expected, a very small percentage (4.9 ± 2.7% in chambers, 3.9 ± 1.3% in loops) of the unstimulated neutrophils (negative control; no infection and no inflammation) released NETs (Fig. 3A, C and 4A; Video S1†). For positive control, a very high increase in percent NET release (64.9 ± 5% in chambers, 66 ± 5.4% in loops) was observed through chemical stimulation with 20 nM PMA (Fig. 3A, C and 4B; Video S1†). Surprisingly, with PAO1 infection, we found a significant increase in the total percentage of neutrophils releasing NETs to PAO1 in the device chambers (12.2 ± 1.8%), but not in loops (6.6 ± 0.8%) (Fig. 3A, C and 4C; Video S1†). In conditions where the neutrophils were stimulated either with the inflammatory cytokines TNF-α [20 ng mL^−1^] or IL-6 [50 ng mL^−1^] in the presence of PAO1, the NET release was significantly higher in both the device chambers and loops. With TNF-α treatment, 27 ± 2.1% of neutrophils released NETs in chambers and 18.6 ± 1.6% in loops (Fig. 3E, F and 4E; Video S1†); and with IL-6 treatment, 23.8 ± 4.7% of neutrophils released NETs in chambers and 13 ± 3.2% in loops (Fig. 3E, F and 4F; Video S1†). Of the two cytokines, TNF-α induced NET release was consistently higher across all the donors (*n* = 8) tested, whereas IL-6 treatment showed individual-to-individual variability. With IL-6 treatment, primary neutrophils from two out of five donors released a significantly higher percentage of NETs but the remaining three donors exhibited a slight increase or no increase in NET release.

On the other hand, treatment with the lipid mediator of inflammation LTB_4_ [20 nM] did not show a significant increase in NET release (19.2 ± 4.3% in chambers, 9.8 ± 1.9% in loops), compared to PAO1 alone (Fig. 3E, F and 4D; Video S1†). While our time-lapse data showed a steep increase in NET release with PMA at 3 h (Fig. 3B and D; Video S1†), NET release to PAO1 (with or without the inflammatory mediators) increased steadily over time (Fig. 3G, H and 4G; Video S1†).

**Fig. 5:**
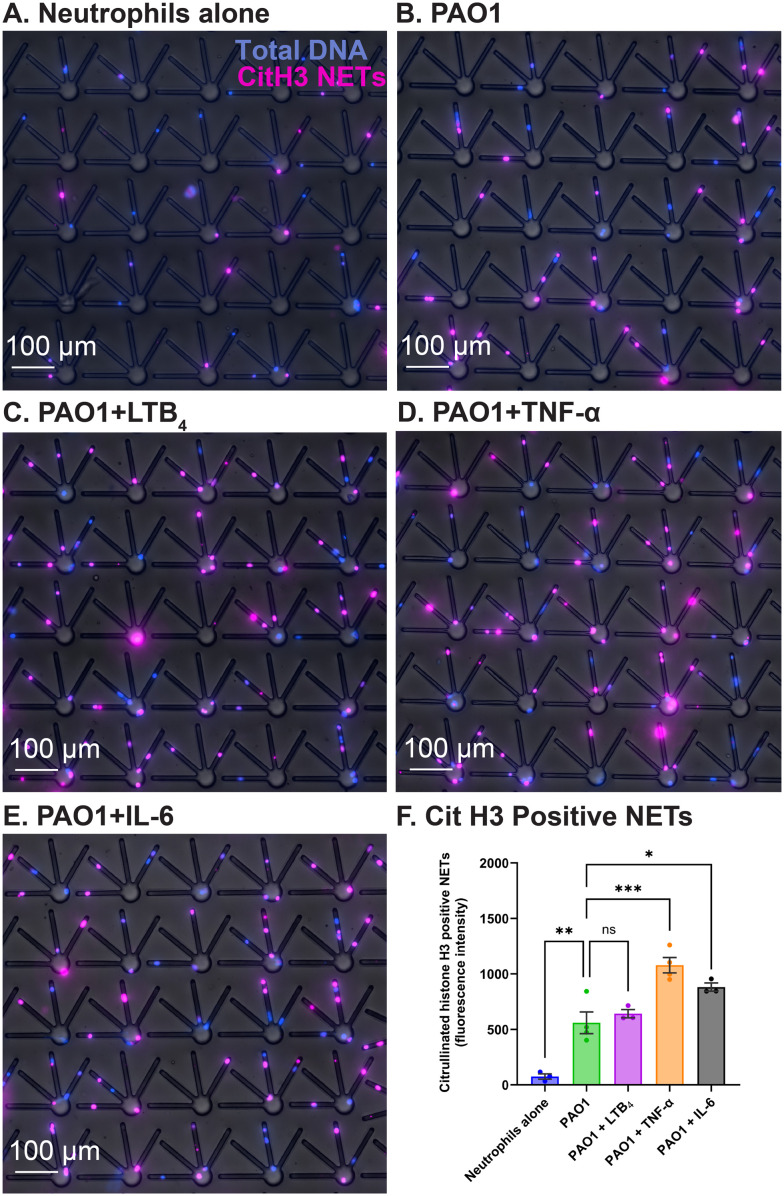
Increased citrullinated histone H3 positive NET release to PAO1 when challenged with the inflammatory cytokines TNF-α and IL-6, measured at the end of the live–dead NETs assay. Blue: total DNA (Hoechst 33342 stained); magenta: CitH3 NETs (anti-citrullinated histone H3 antibody stained). (A) Neutrophils alone (B) neutrophils + PAO1 (no inflammatory mediator) (C) neutrophils + PAO1 + 20 nM LTB_4_ (D) neutrophils + PAO1 + 20 ng mL^−1^ TNF-α and (E) neutrophils + PAO1 + 50 ng mL^−1^ IL-6. (F) Total fluorescence intensity values of citrullinated histone H3 across the NETs released (from *N* = 200 neutrophils; chambers and loops combined) in each of the treatment groups compared to the negative control (neutrophils without PAO1 and inflammatory mediators). ns – not significant, *p < 0.05, **p < 0.01, ****p < 0.0001; one-way ANOVA with Dunnett's multiple comparisons test used. Data are represented as mean ± SEM and collected from 3 separate healthy volunteers.

We also demonstrated the heterogeneity of proteins on NETs by immunofluorescent labeling citrullinated histone H3, a NET release marker. We computed the fluorescence intensity values of citrullinated histone H3 from NETs released during the live–dead NETs assay, showing increased citrullinated histone H3 positive NET release to PAO1 when challenged with the inflammatory cytokines TNF-α and IL-6 (Fig. 5).

**Fig. 6:**
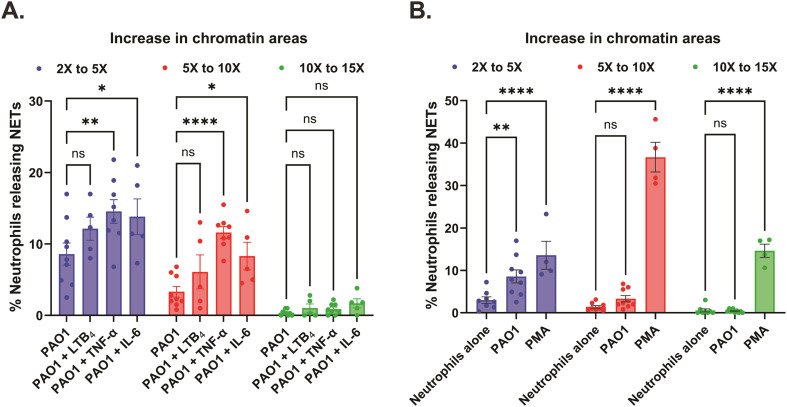
Increased percentage of variable size NET release with PMA and combined PAO1 – inflammatory mediator treatment, in the device chambers. Data are represented as mean ± SEM and collected from 5 to 9 separate healthy volunteers, except for positive control where *n* = 4 donors. 20 nM PMA was used as the positive control to stimulate NET release, while neutrophils (without PAO1 and inflammatory mediators) were used as the negative control in the assay. (A) Graph comparing the distribution of the relative areas of NETs released to PAO1 with or without the inflammatory mediators 20 nM LTB_4_, 20 ng mL^−1^ TNF-α and 50 ng mL^−1^ IL-6 respectively, in the device chambers. ns – not significant, *p < 0.05, **p < 0.01, ****p < 0.0001; two-way ANOVA with Tukey's multiple comparisons test used. (B) Graph showing the distribution of the relative areas of NETs with PMA [20 nM] or PAO1 compared to the negative control, in the device chambers. ns – not significant, *p < 0.05, **p < 0.01, ****p < 0.0001; two-way ANOVA with Tukey's multiple comparisons test used.

Further probing into the spatial dynamics of the NET release in the chambers of our engineered microfluidic system, we measured the areas of the decondensed, diffused chromatin of the individual dead neutrophils (confirmed with Sytox orange – dead cell DNA stain) and the area of NETs was calculated relative to the area of live nuclei (pre-stained with Hoechst – live cell DNA stain). We based all our comparative analyses of NET release considering an increase in the decondensed chromatin areas of the dead neutrophils by a factor greater than or equal to 2, to classify them as NETs. Accordingly, we categorized the increase in chromatin areas to values ranging from 2× to 5×, 5× to 10×, and 10× to 15× respectively. In response to PAO1 with or without the inflammatory mediator treatment, we found that most neutrophils released NETs in the 2× to 5× range (8.6 ± 1.5% with PAO1, 12.1 ± 1.6% with PAO1 + LTB_4_, 14.5 ± 1.7% with PAO1 + TNF-α, and 13.8 ± 2.5% with PAO1 + IL-6), followed by NETs in the 5× to 10× range (3.3 ± 0.7% with PAO1, 6.1 ± 2.4% with PAO1 + LTB_4_, 11.6 ± 0.8% with PAO1 + TNF-α, and 8.3 ± 1.9% with PAO1 + IL-6), and almost negligible percentage of NETs in the 10× to 15× range (0.4 ± 0.1% with PAO1, 1 ± 0.6% with PAO1 + LTB_4_, 0.9 ± 0.3% with PAO1 + TNF-α, and 1.7 ± 0.6% with PAO1 + IL-6) (Fig. 6A). Although we report that overall neutrophils release a higher percentage of NETs in the 2× to 5× range with combined PAO1 – inflammatory mediator treatment (TNF-α/IL-6), it is important to note that these neutrophils also release significantly higher percentage of NETs in 5× to 10× range, compared to infection without inflammation (Fig. 6A – center panel). Neutrophils when treated with PMA displayed a complete reversal of the trend, with most neutrophils releasing NETs in the 5× to 10× range (36.7 ± 3.5%), followed by an almost equal percentage of neutrophils releasing NETs in the 10× to 15× range (14.6 ± 1.6%) and 2× to 5× range (13.6 ± 3.3%) (Fig. 6B).

### Comparative analysis of NET release in the confined side loops and wide chambers of the NET-array device

**Fig. 7:**
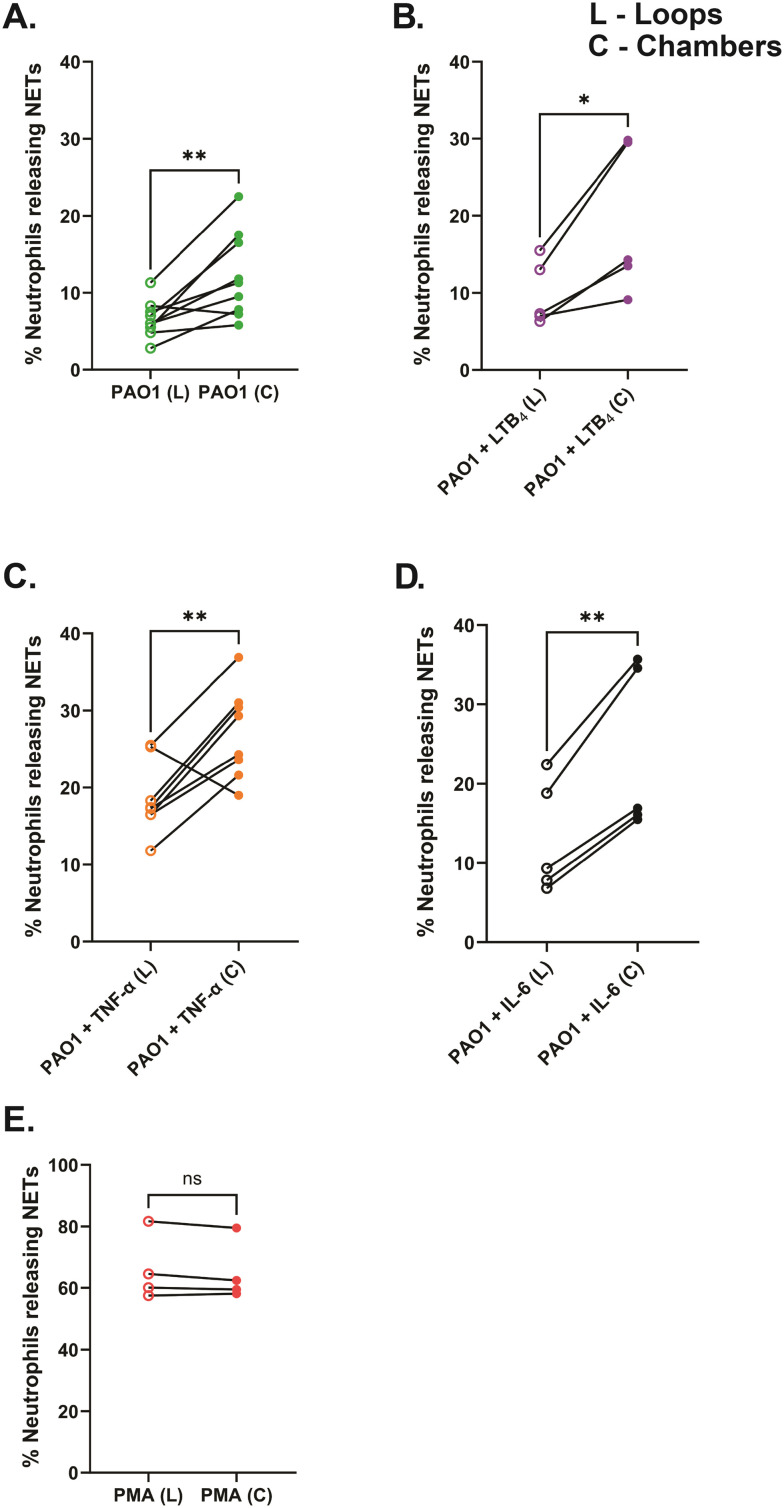
Neutrophils release fewer NETs to *P. aeruginosa* (PAO1) in the confined loops (L) compared to the relatively wider chambers (C) of the device, with or without the inflammatory mediators. (A) Neutrophils + PAO1 (no inflammatory mediator) (B) neutrophils + PAO1 + 20 nM LTB_4_ (C) neutrophils + PAO1 + 20 ng mL^−1^ TNF-α and (D) neutrophils + PAO1 + 50 ng mL^−1^ IL-6 (E) positive control (20 nM PMA). Data are represented as mean ± SEM and collected from 5 to 9 separate healthy volunteers, except for positive control where *n* = 4 donors. ns – not significant, *p < 0.05, **p < 0.01; two-tailed paired student *t*-test used.

To quantify NET-release in variably confined spaces, we incorporated wide chambers (42 μm diameter) and narrow side loops (2 μm width) in our NET-array wells. We quantified the NET release from primary healthy human neutrophils trapped in the narrow loops *versus* the relatively wider chambers of the device across different treatment conditions. We found that the neutrophils trapped in the confined loops of the device released fewer NETs across all the conditions including PAO1 with or without the inflammatory mediator in the microenvironment (6.6 ± 0.8% with PAO1, 9.8 ± 1.9% with PAO1 + LTB_4_, 18.6 ± 1.6% with PAO1 + TNF-α, and 13 ± 3.2% with PAO1 + IL-6, respectively), compared to the neutrophils in the relatively wider chambers (12.2 ± 1.8% with PAO1, 19.2 ± 4.3% with PAO1 + LTB_4_, 27 ± 2.1% with PAO1 + TNF-α, and 23.8 ± 4.7% with PAO1 + IL-6, respectively) (Fig. 7A–D). However, with PMA treatment, a very high percentage of neutrophils released NETs equally in both the loops (66 ± 5.4%) and chambers (64.9 ± 5%) of the device, with no significant difference (Fig. 7E). We also observed that the NETs released in loops plugged the confined spaces (Fig. 2B, 4 and 5; Video S1†).

Further, we wanted to verify that the lower percentage of NET release in the loops compared to chambers is not a result of the containment of the chromatin diffusion from NETs in the loops. To validate this, we ran a comparative analysis of the total dead neutrophil percent in the loops and chambers of the device, quantified by dead cell staining (we used Sytox orange in our study). We found a similar trend as reported previously with NETs, showing reduced dead neutrophil percent in the loops (9.6 ± 1.5% with PAO1, 12 ± 2.1% with PAO1 + LTB_4_, 23.2 ± 1.6% with PAO1 + TNF-α, and 16.6 ± 4% with PAO1 ± IL-6, respectively), compared to the neutrophils in the chambers (17.2 ± 2.8% with PAO1, 23.5 ± 5% with PAO1 + LTB_4_, 32.4 ± 2.6% with PAO1 + TNF-α, and 29.1 ± 6% with PAO1 ± IL-6, respectively) (Fig. S3A–D†). PMA-treated neutrophils still showed similar cell death (71.5 ± 5.2% in loops and 75.5 ± 5% in chambers) (Fig. S3E†).

## Discussion

The NET-array device developed and validated in this study, enabled the quantification of increased NET release to *Pseudomonas aeruginosa* PAO1 bacterial infection in microenvironments rich in inflammatory cytokines, TNF-α and IL-6. Our findings align with the current literature showing that TNF-α and IL-6 increase NET release *in vitro*.^58^ In our NET-array system, we incorporated *P. aeruginosa* in addition to inflammatory mediators to quantify the cumulative effects of infection and inflammation on NET release. We showed that primary healthy human neutrophils challenged with TNF-α or IL-6 release NETs to a greater extent compared to PAO1 alone. This underpins the importance of immune-modulating anti-cytokine treatments in the clinic for patients with elevated levels of TNF-α and IL-6 in serum to serve better prognosis in inflammatory conditions like sepsis^31^ and COVID-19.^32,33,59^ Surprisingly, we found that NET release is unaffected when treated with LTB_4_, a significant lipid mediator of inflammation, previously shown to increase neutrophil recruitment and swarming *in vivo*.^7,60^ However, the influence of LTB_4_ on NET release, especially in the context of infection, has not been previously explored.

Our research further investigates the influence of neutrophil extracellular traps (NETs) within restricted environments, analogous to those found in microvascular networks. We investigated if the mechanical stress and cell stretching causing nuclear deformation during the squeezing of neutrophils in confined spaces^61–63^ potentially influenced NET release dynamics, in a combined infection–inflammation microenvironment. This is relevant because of the highly regulated sequence of events leading to nuclear decondensation during NET release, possibly affected by mechanical perturbations. We found reduced NET release from primary healthy human neutrophils confined in the side loops compared to the relatively wider chambers in the device, in either the treatment conditions [PAO1 – inflammatory mediator (TNF-α/IL-6)] or with PAO1 alone. This observation, along with the quantification of variable NET sizes, underscores the heterogeneity of NET formation pathways. This is important to study because it is shown that NETs have the ability to perturb the blood flow by mechanically obstructing micro-channel networks *in vitro*, thereby providing insights into the detrimental effect of NETs in confined microenvironments.^64^ It is also known that neutrophils *en route* to the site of infection traverse through spatially restricted environments including microvascular lumen and extravascular tissue spaces *in vivo*.^65^ Further probing into the spatial dynamics of NET release, we quantified the differences in the relative areas of NETs and reported a high percentage of variable size NET release in our NET-array device. There is an increasing body of literature documenting the heterogeneity of NET formation pathways (suicidal and non-lytic NET release, PAD-dependent and -independent, TLR2/1-dependent and independent),^14,15,66^ and in the future, our NET-array system can facilitate research to unravel the mechanistic links between NET size, formation pathways, and the modulation of NET release, potentially guiding therapeutic interventions in inflammatory diseases. The early successes with the characterization of the NET formation pathways may then be tailored to regulate NET release and curtail inflammation without compromising the NET-mediated host defense.

Quantifying the dynamics of NET release using the NET-array device will pave the way for effective methods to assess the level of inflammation and neutrophil responses to infections in patients. Our NET-array system works to delineate the complex signaling schema involved in NET release dynamics at a single-cell resolution, using a bottom-up approach dissecting the individual role of the predominant inflammatory mediators LTB_4_, TNF-α, and IL-6 in mediating NET release. This data can be utilized to create a quantitative framework to better understand the interplay between infection and inflammation. Advanced engineering-driven approaches/microtechnologies better recapitulating the *in vivo* 3D confined spaces may be essential to further investigate the role of mechanical confinement on NET release. These studies probing into the NET release paradigm against infections in patients with inflammatory conditions like sepsis could give us more information on the effect of the mechanochemical complexity of the microenvironment on NET release in patients. This knowledge could open avenues for developing targeted interventions aimed at fine-tuning NET release, thereby mitigating the harmful effects of excessive NET release and promoting better disease management and treatment outcomes. This platform may enable investigation into the possible effects of immune-modulating therapies in clinical settings.

## Author contributions

Udaya Sree Datla led the study, conducted all experiments, and wrote the manuscript. Bhaskar Vundurthy helped in designing the device and played a key role in developing and running MATLAB simulations for the automated image analysis. Jessica Hook and Dr. Jessica Moreland, M.D. played an instrumental role in healthy volunteer enrollment and blood sample collection at The University of Texas Southwestern Medical Center. Hossein Razmi Bagtash helped with image analysis. Nidhi Menon, Tarik Shihabeddin and Dr. David Schmidtke helped set up device fabrication. Dr. Marko Radic advised on experimental design. Dr. Caroline N. Jones is the principal investigator who conceived, directed, and funded the study. All the authors took an active part in reviewing the manuscript.

## Conflicts of interest

The authors declare no conflicts of interest.

## Supplementary Material

LC-024-D3LC00648D-s001

LC-024-D3LC00648D-s002

LC-024-D3LC00648D-s003
