# Supplementary figures and images for: Quantifying neutrophil extracellular trap release in a combined infection–inflammation NET-array device
Source: Lab Chip. 2024 Jan 4;24(3):615–28. doi: 10.1039/d3lc00648d (PMC10826461; doi:10.1039/d3lc00648d)

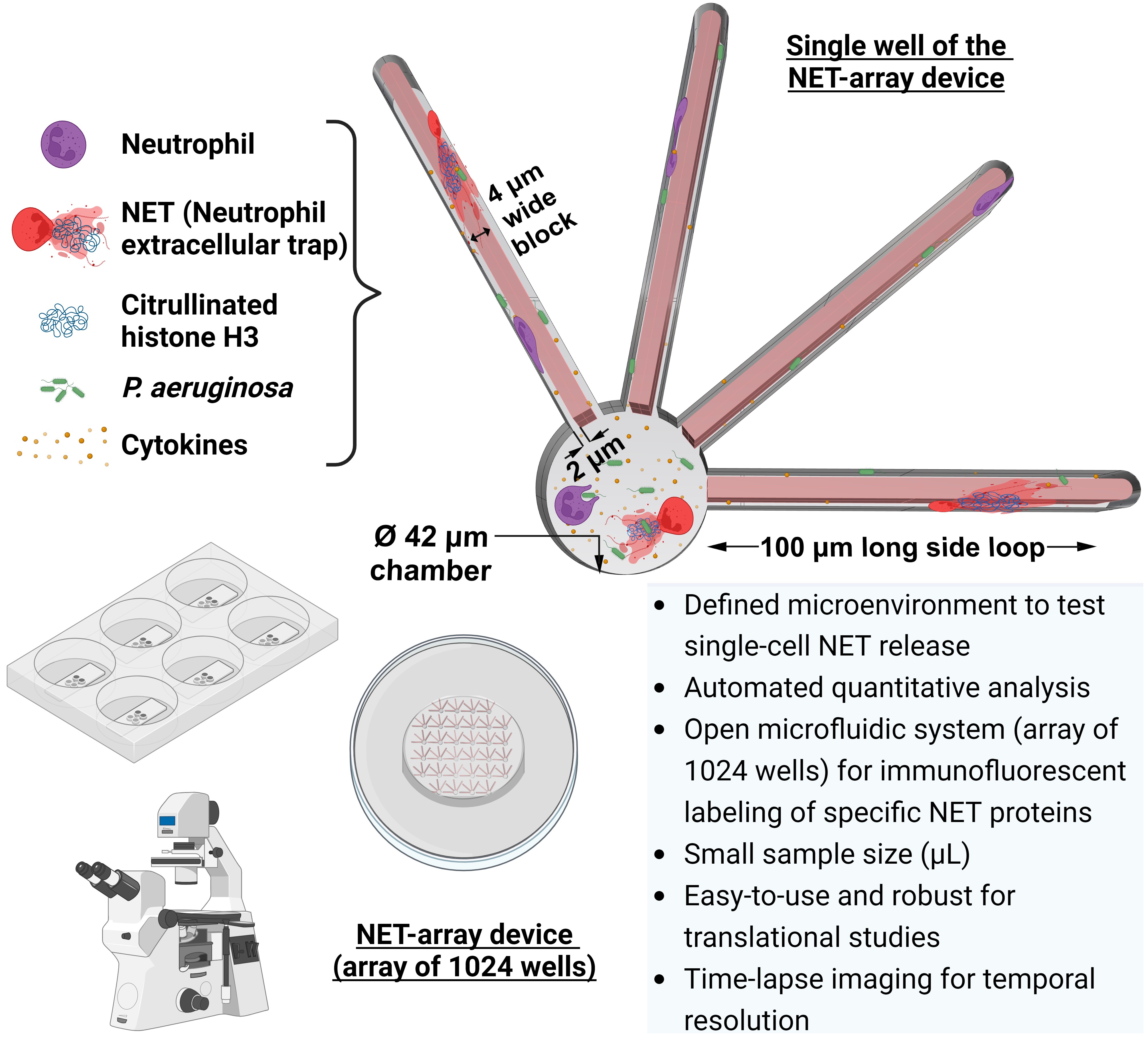

Supplement: LC-024-D3LC00648D-s001 [file LC-024-D3LC00648D-s001.zip › Manuscript figures Jpg/Fig. 1.jpg]

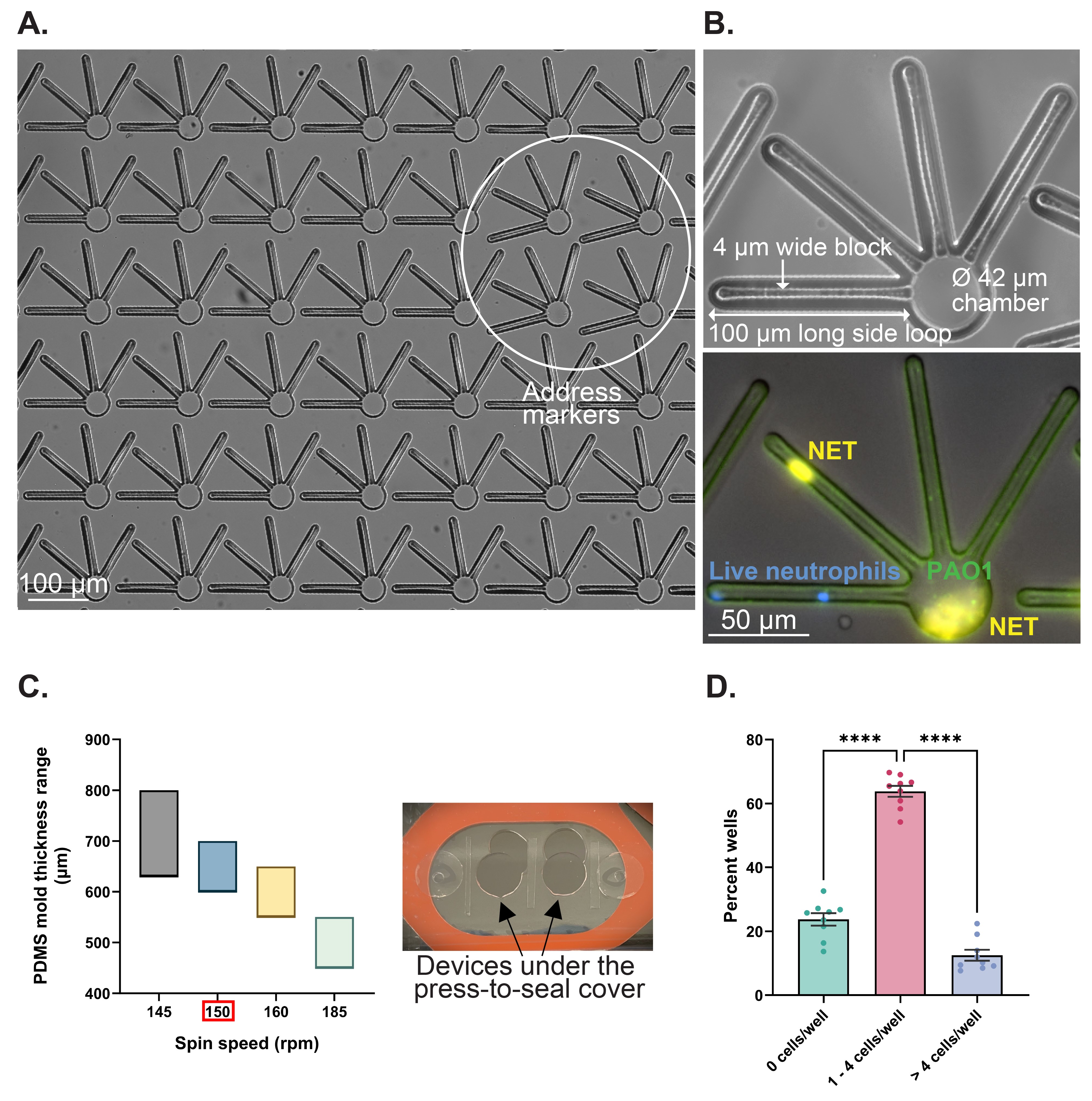

Supplement: LC-024-D3LC00648D-s001 [file LC-024-D3LC00648D-s001.zip › Manuscript figures Jpg/Fig. 2.jpg]

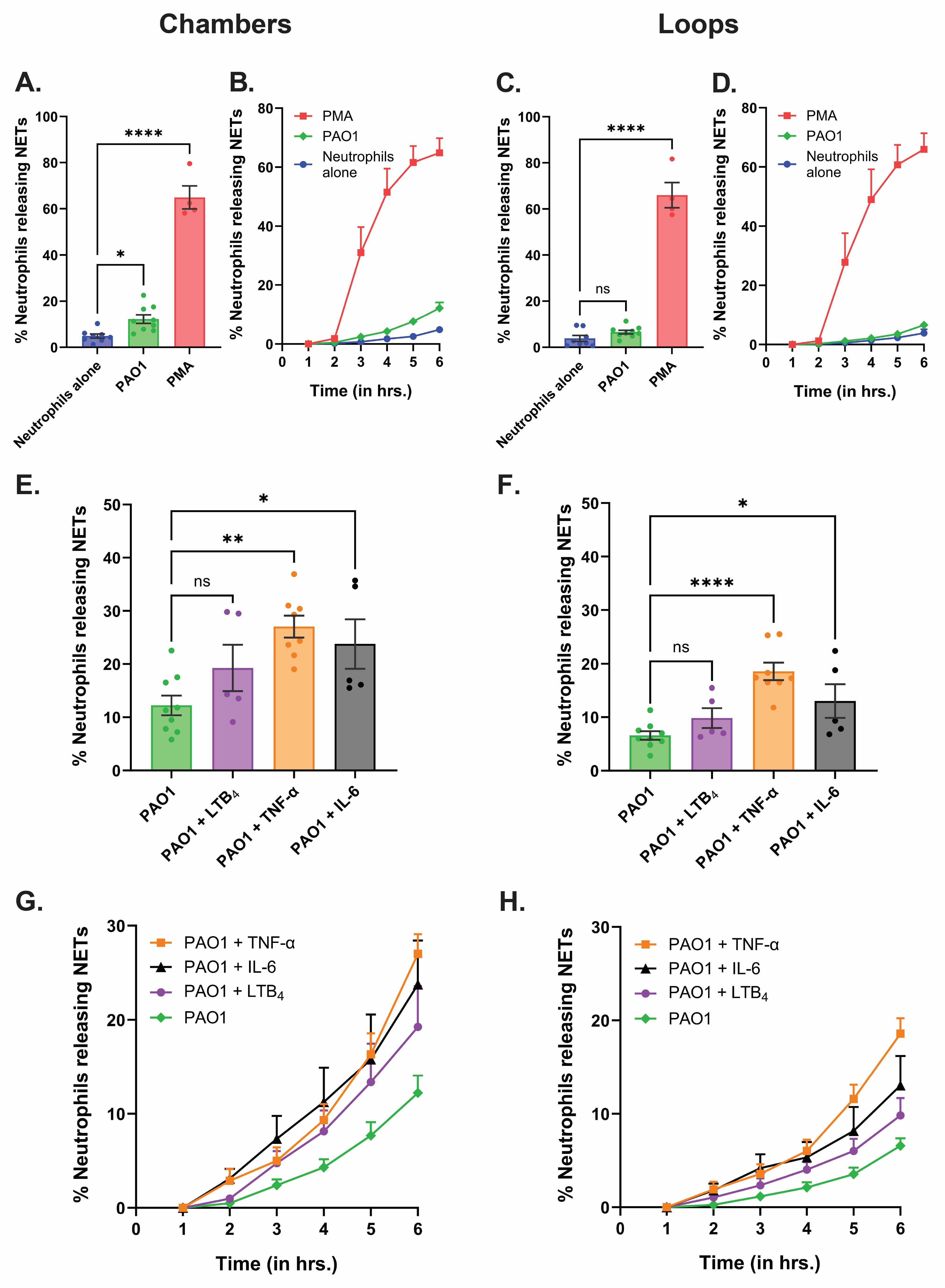

Supplement: LC-024-D3LC00648D-s001 [file LC-024-D3LC00648D-s001.zip › Manuscript figures Jpg/Fig. 3.jpg]

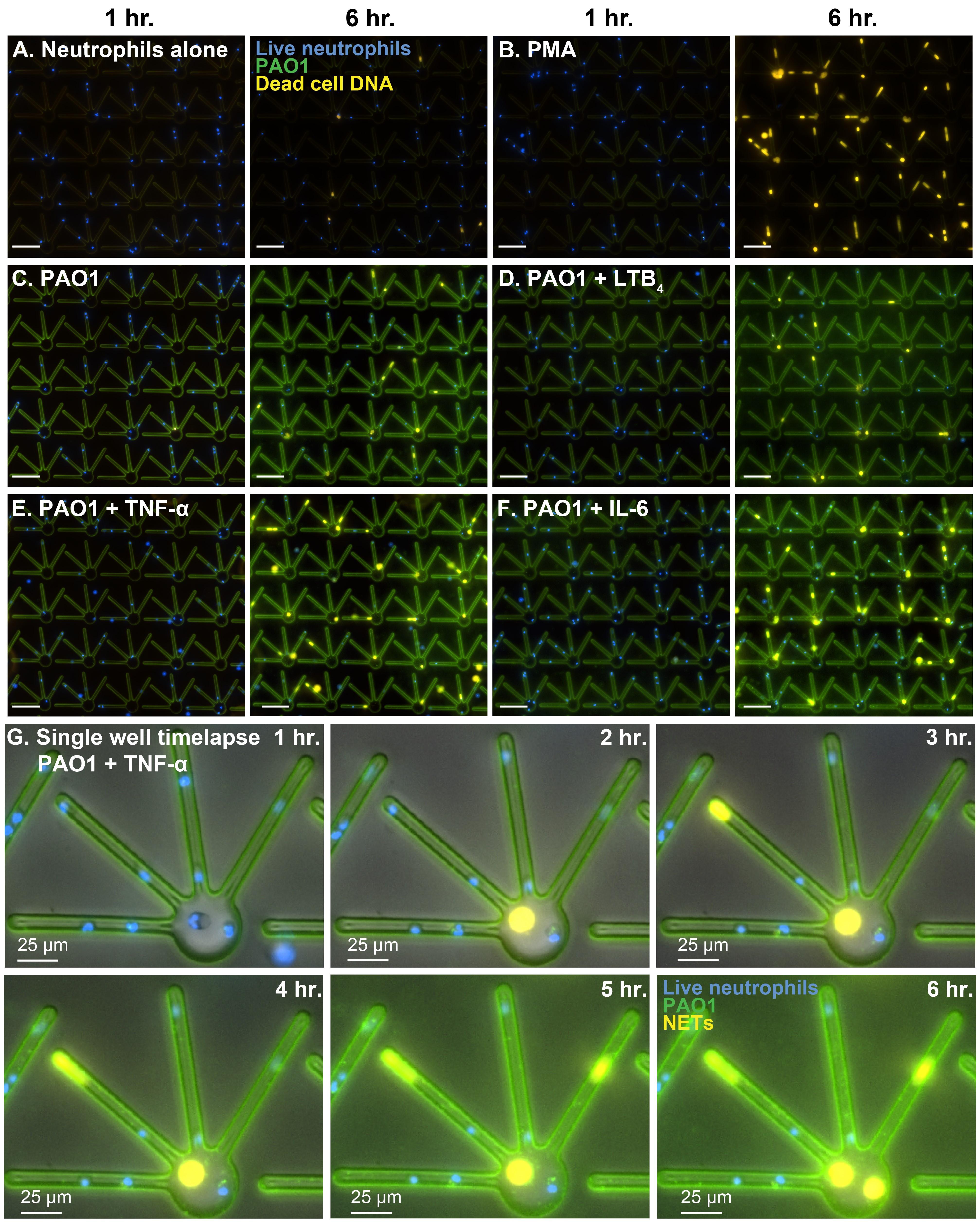

Supplement: LC-024-D3LC00648D-s001 [file LC-024-D3LC00648D-s001.zip › Manuscript figures Jpg/Fig. 4.jpg]

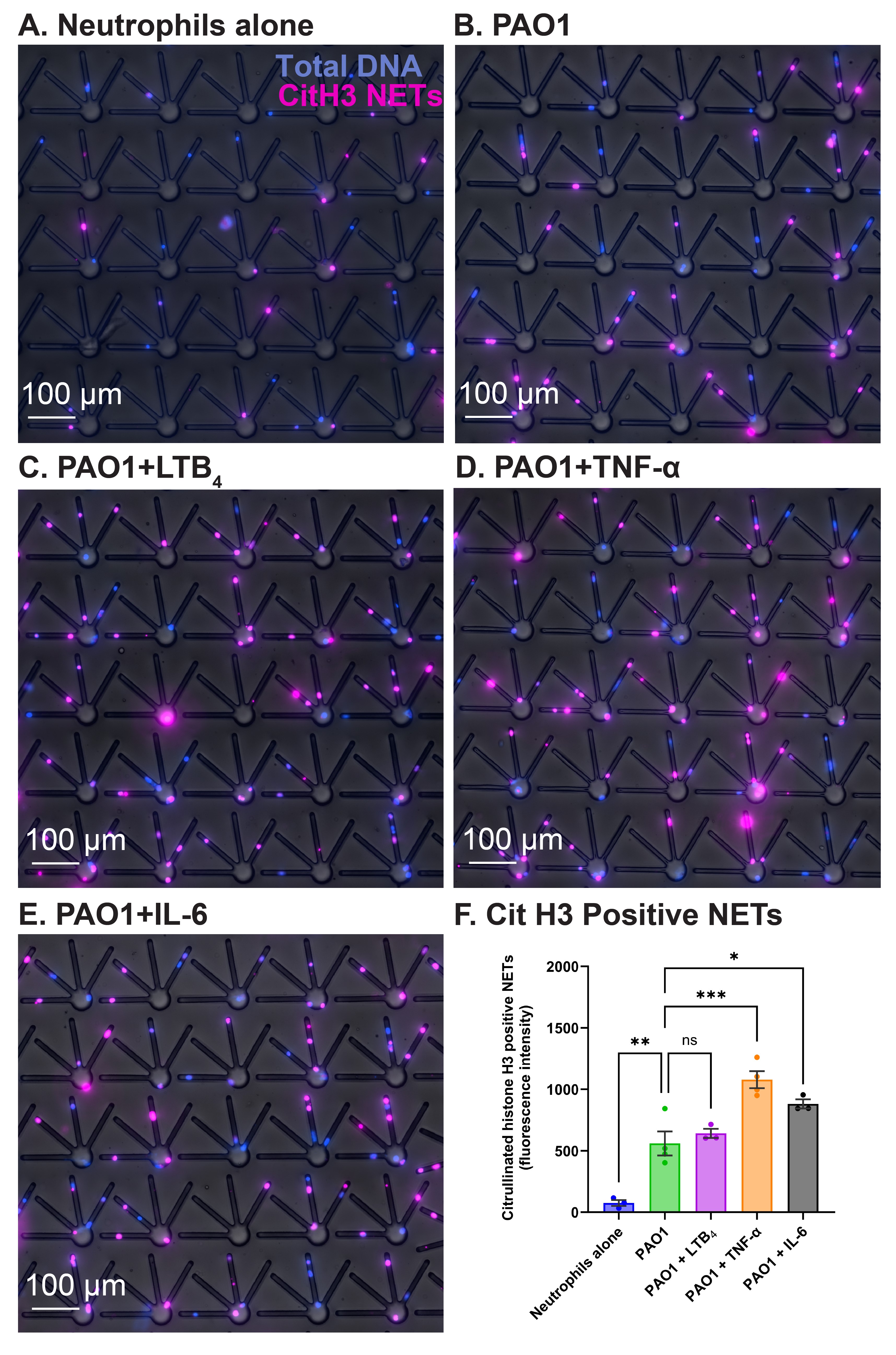

Supplement: LC-024-D3LC00648D-s001 [file LC-024-D3LC00648D-s001.zip › Manuscript figures Jpg/Fig. 5.jpg]

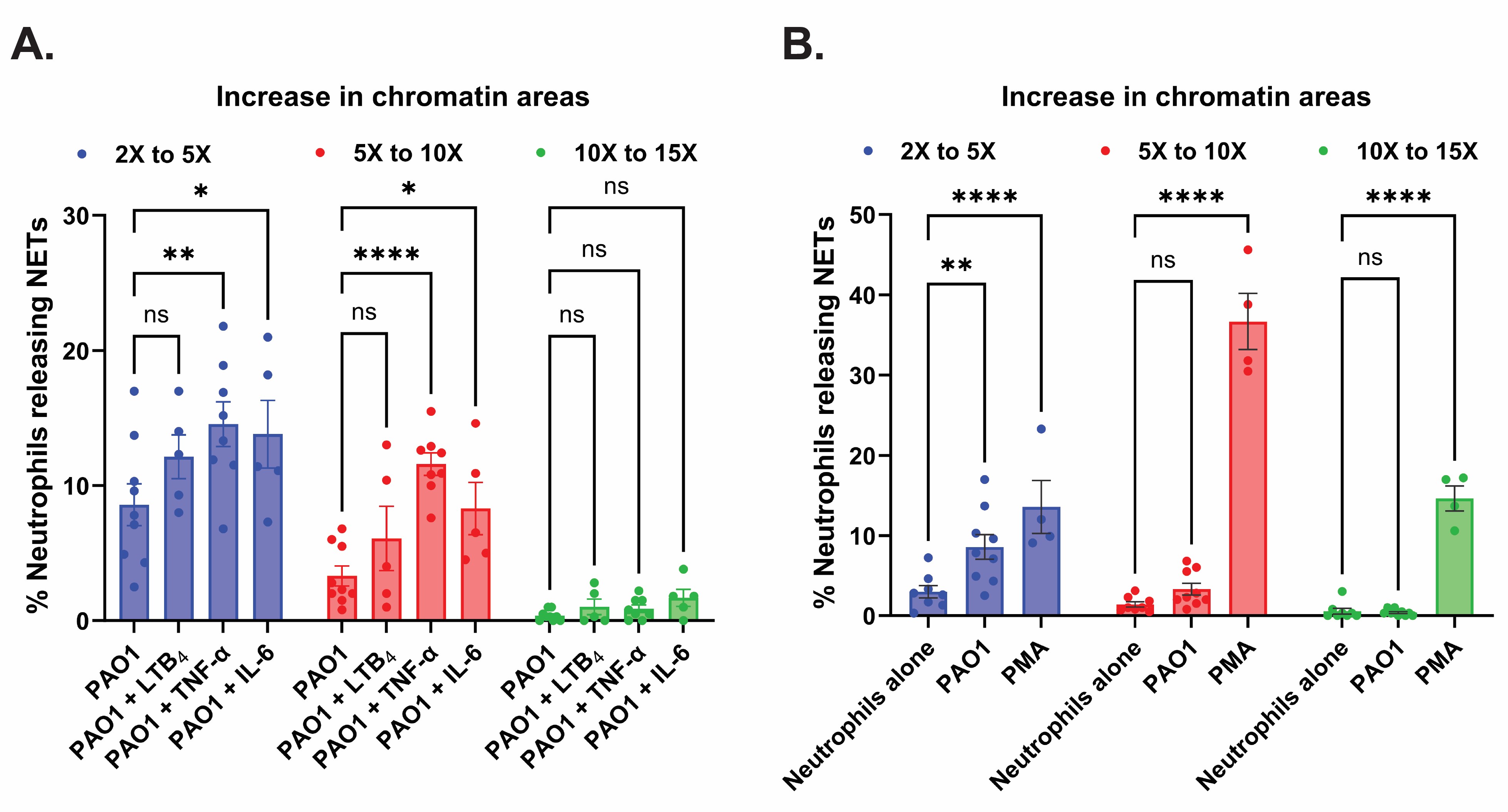

Supplement: LC-024-D3LC00648D-s001 [file LC-024-D3LC00648D-s001.zip › Manuscript figures Jpg/Fig. 6.jpg]

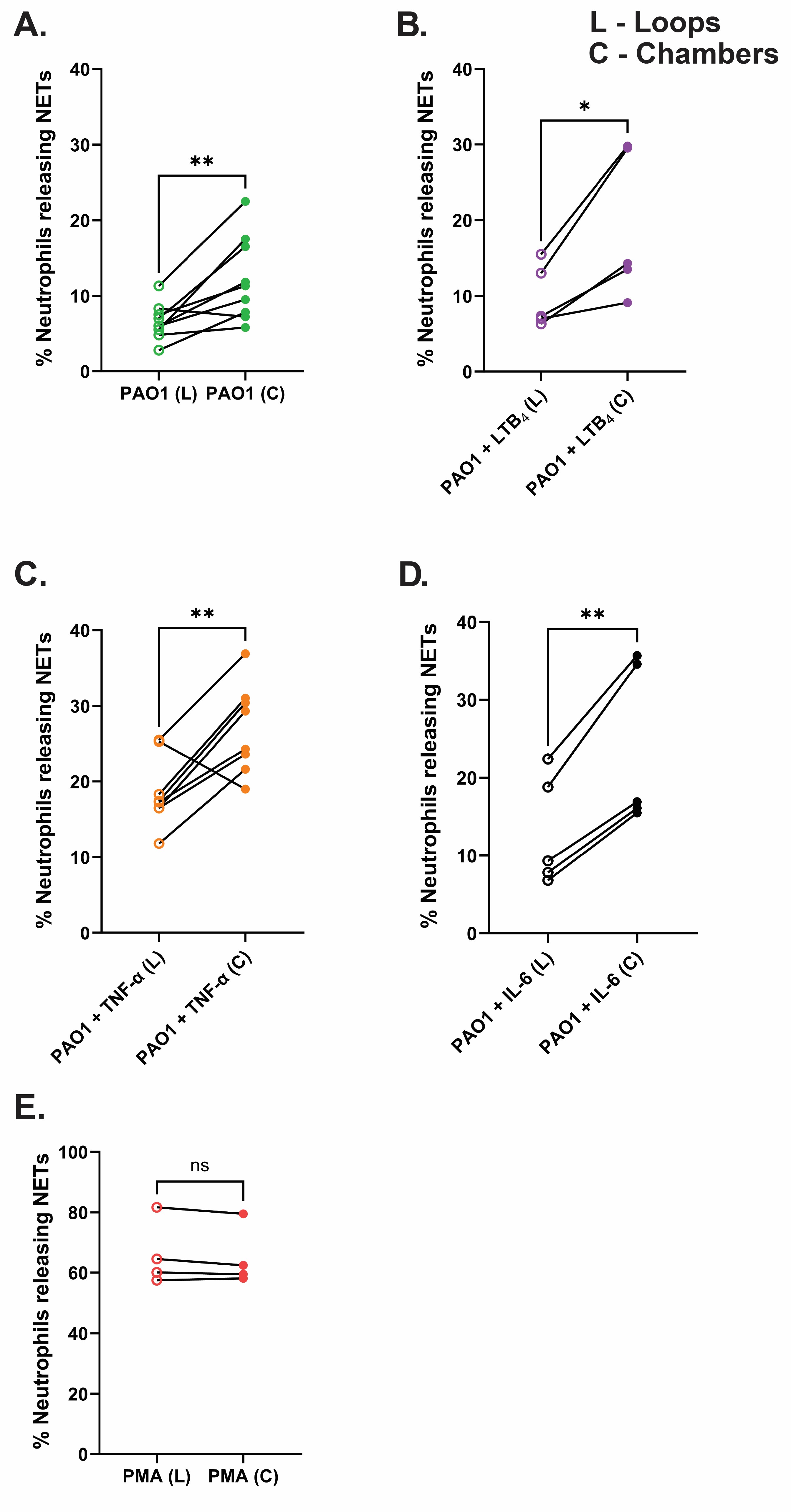

Supplement: LC-024-D3LC00648D-s001 [file LC-024-D3LC00648D-s001.zip › Manuscript figures Jpg/Fig. 7.jpg]

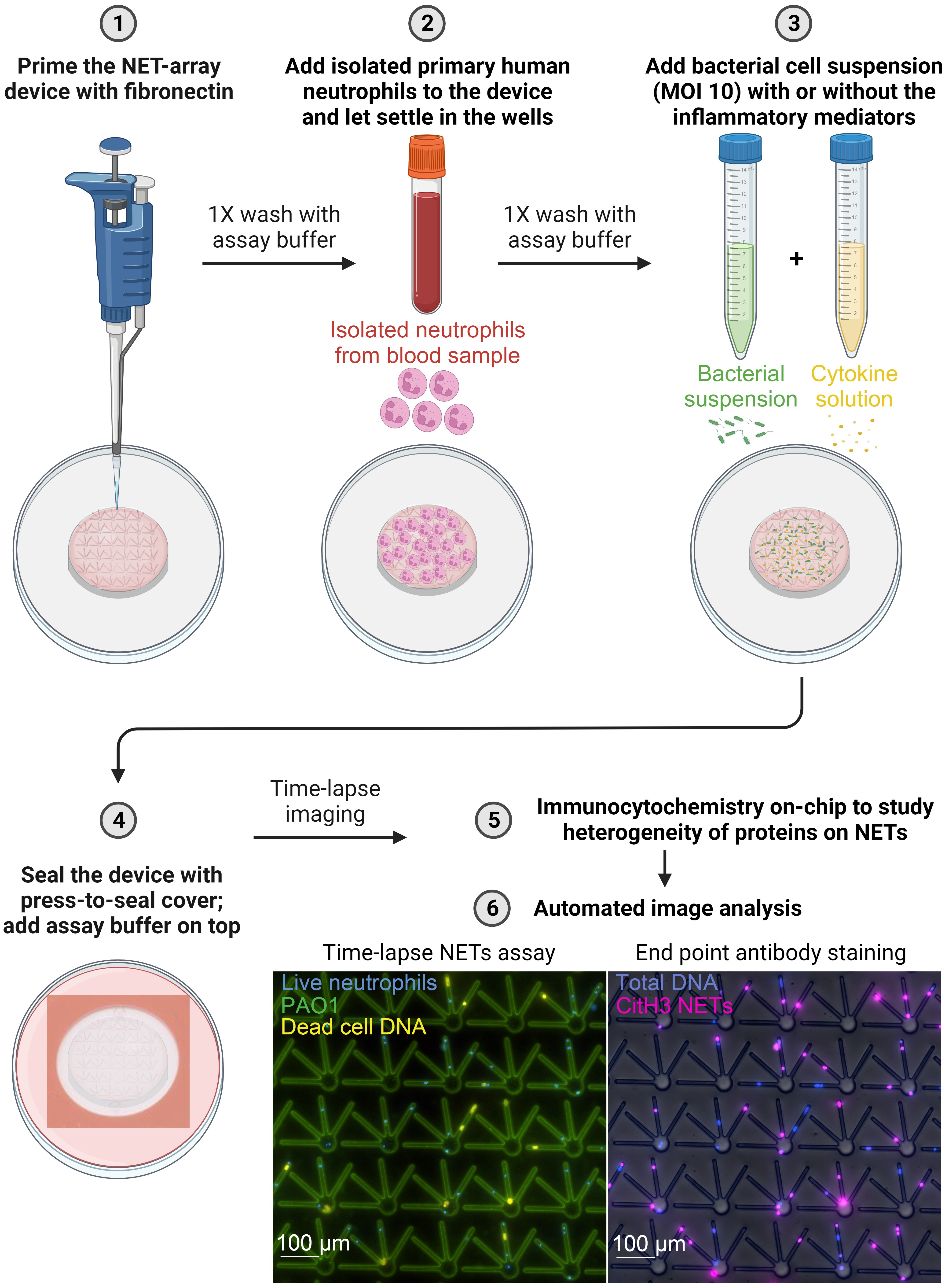

Supplement: LC-024-D3LC00648D-s001 [file LC-024-D3LC00648D-s001.zip › Manuscript figures Jpg/Fig. S1.jpg]

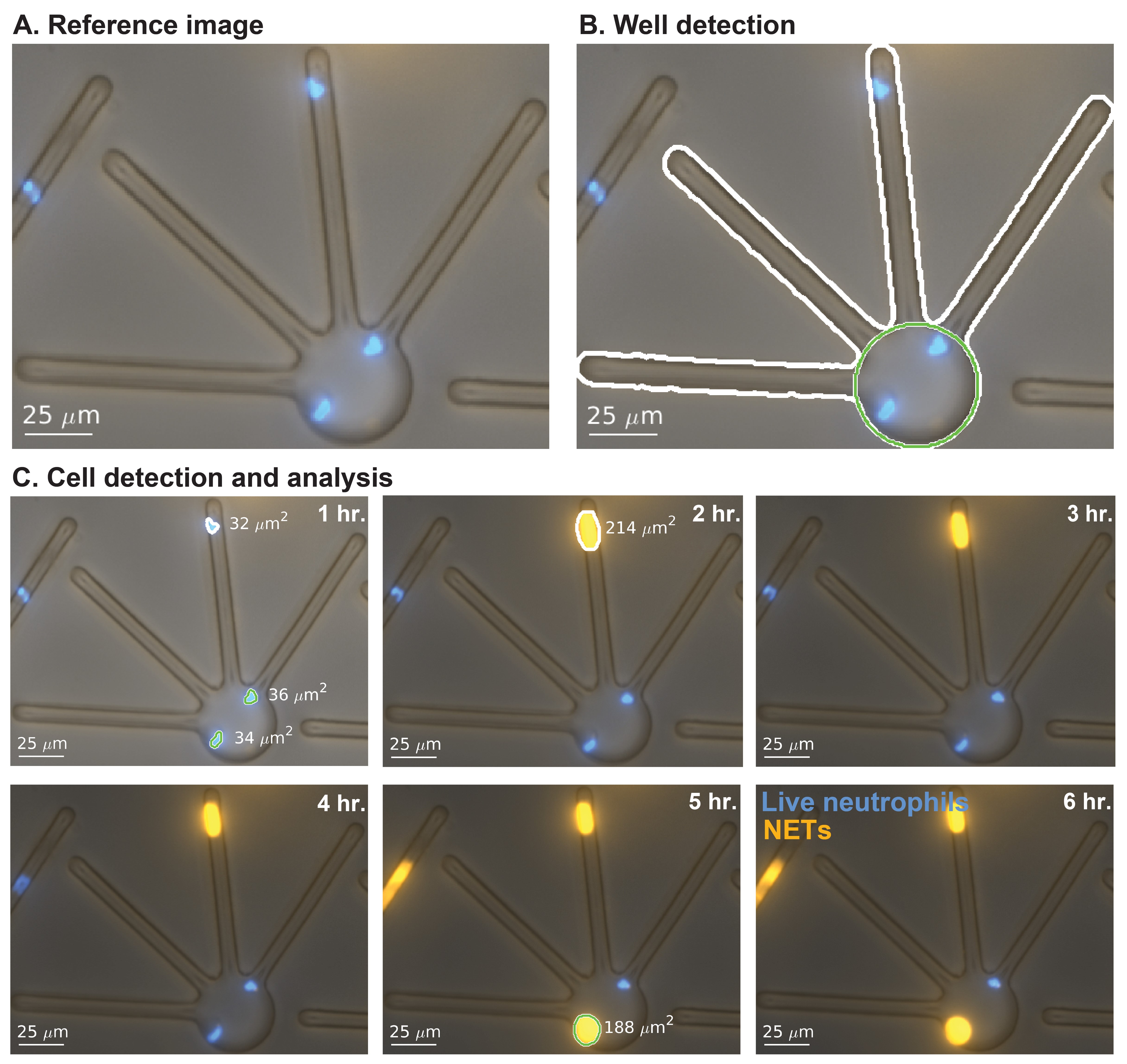

Supplement: LC-024-D3LC00648D-s001 [file LC-024-D3LC00648D-s001.zip › Manuscript figures Jpg/Fig. S2.jpg]

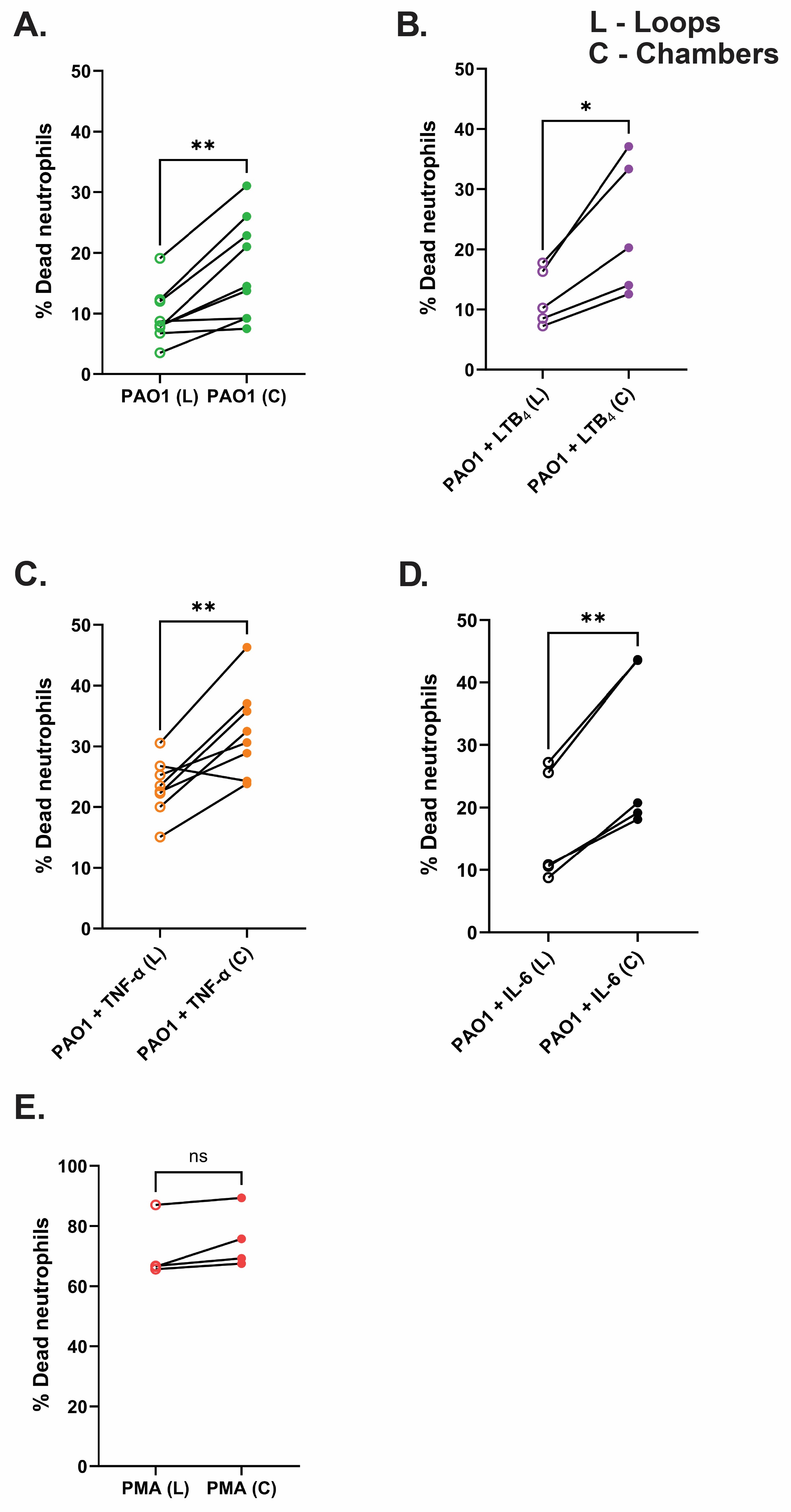

Supplement: LC-024-D3LC00648D-s001 [file LC-024-D3LC00648D-s001.zip › Manuscript figures Jpg/Fig. S3.jpg]

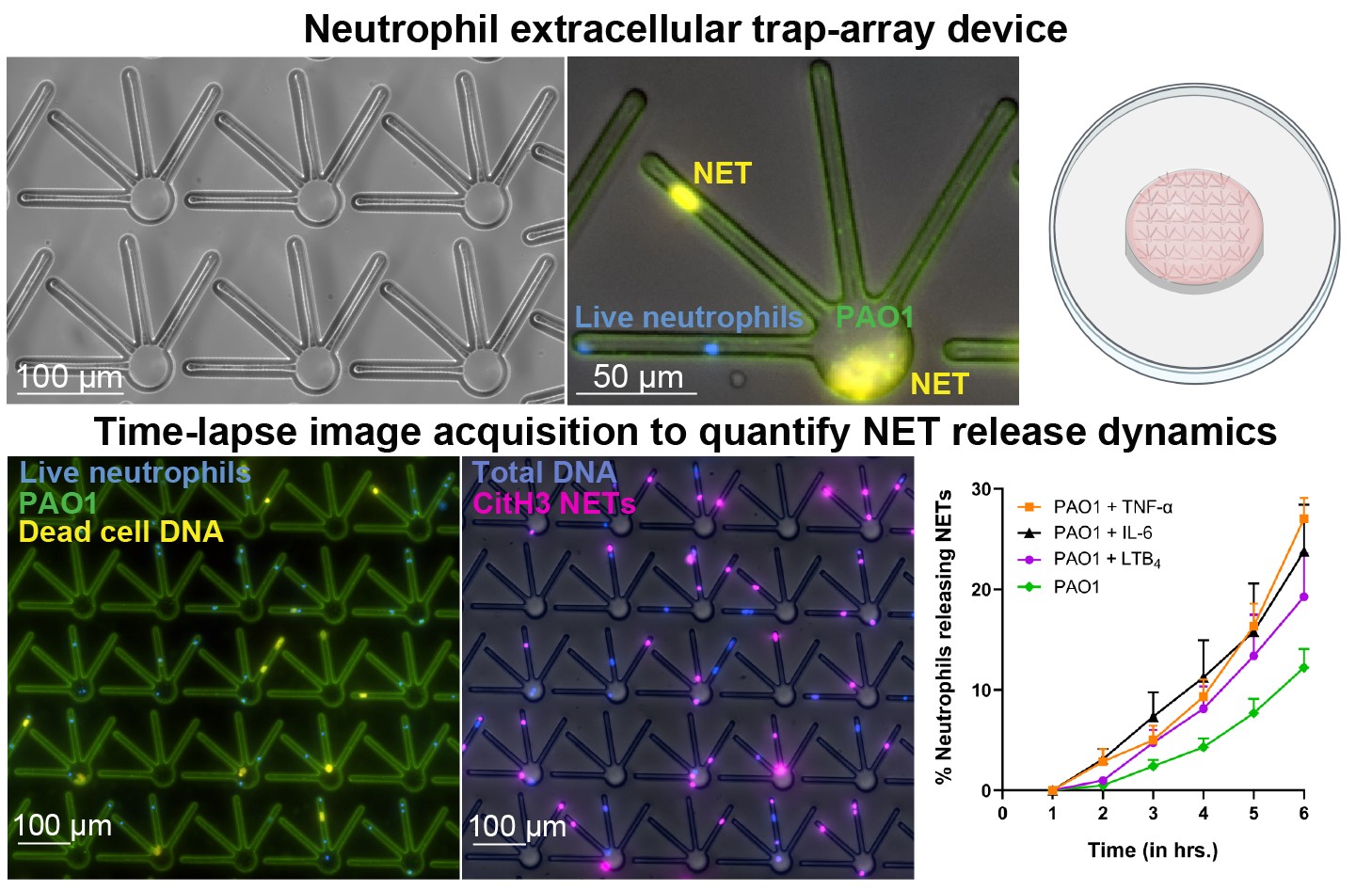

Supplement: LC-024-D3LC00648D-s001 [file LC-024-D3LC00648D-s001.zip › Manuscript figures Jpg/Graphical abstract.jpg]
