# Supplementary material for: Quantifying neutrophil extracellular trap release in a combined infection–inflammation NET-array device
Source: Lab Chip. 2024 Jan 4;24(3):615–28. doi: 10.1039/d3lc00648d (PMC10826461; doi:10.1039/d3lc00648d)
Supplement: LC-024-D3LC00648D-s002 [file LC-024-D3LC00648D-s002.pdf]

Electronic Supplementary Material (ESI) for Lab on a Chip.
This journal is © The Royal Society of Chemistry 2024

Quantifying Neutrophil Extracellular Trap Release in a Combined Infection-Inflammation NET-Array Device

Udaya Sree Datla[1,2,3], Bhaskar Vundurthy[4], Jessica S. Hook[5], Nidhi Menon[1], Hossein Razmi Bagtash[2,3], Tarik Shihabeddin[2,3], David W. Schmidtke[2,3], Jessica G. Moreland[5,6], Marko Z. Radic[7], Caroline N. Jones[†2,3].

1. Translational Biology, Medicine and Health, Virginia Polytechnic Institute and State University, Blacksburg, VA, USA.
2. Department of Bioengineering, University of Texas at Dallas, Richardson, TX, USA.
3. Department of Surgery, University of Texas Southwestern medical center, Dallas, TX, USA.
4. Robotics Institute, Carnegie Mellon University, Pittsburgh, PA, USA.
5. Department of Pediatrics, University of Texas Southwestern medical center, Dallas, TX, USA.
6. Department of Microbiology, University of Texas Southwestern medical center, Dallas, TX, USA.
7. Department of Microbiology, Immunology and Biochemistry, University of Tennessee Health Science Center, Memphis, TN, USA.

[†] Corresponding author: Caroline N. Jones, electronic address: Caroline.jones@utdallas.edu

Keywords: Neutrophil extracellular traps, NET-array device, *Pseudomonas aeruginosa*, Infection, Inflammation

Supplementary Information

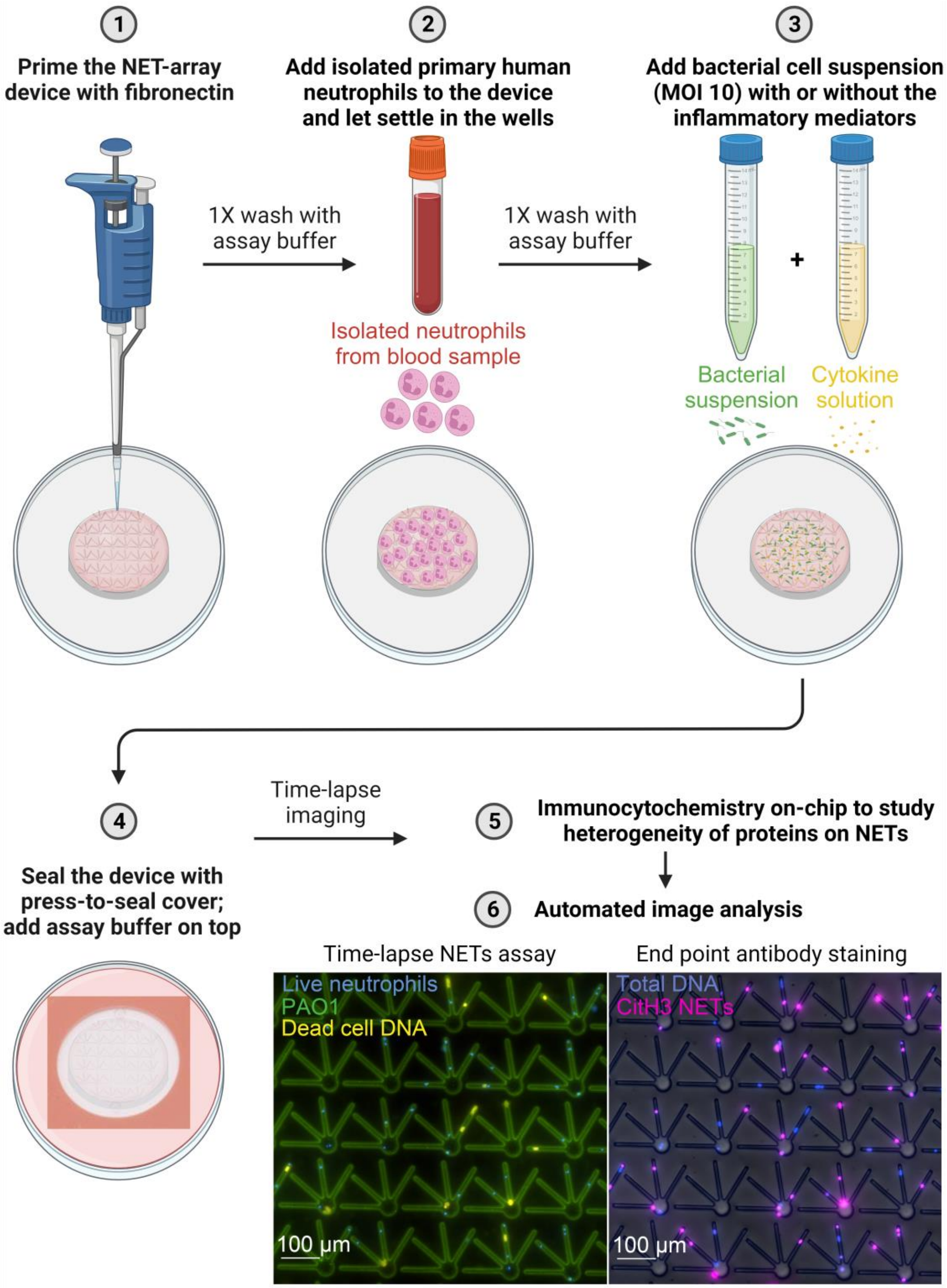

Figure S1. Detailed schematic of the experimental workflow developed to quantify NET release dynamics in the combined infection-inflammation NET-array device.

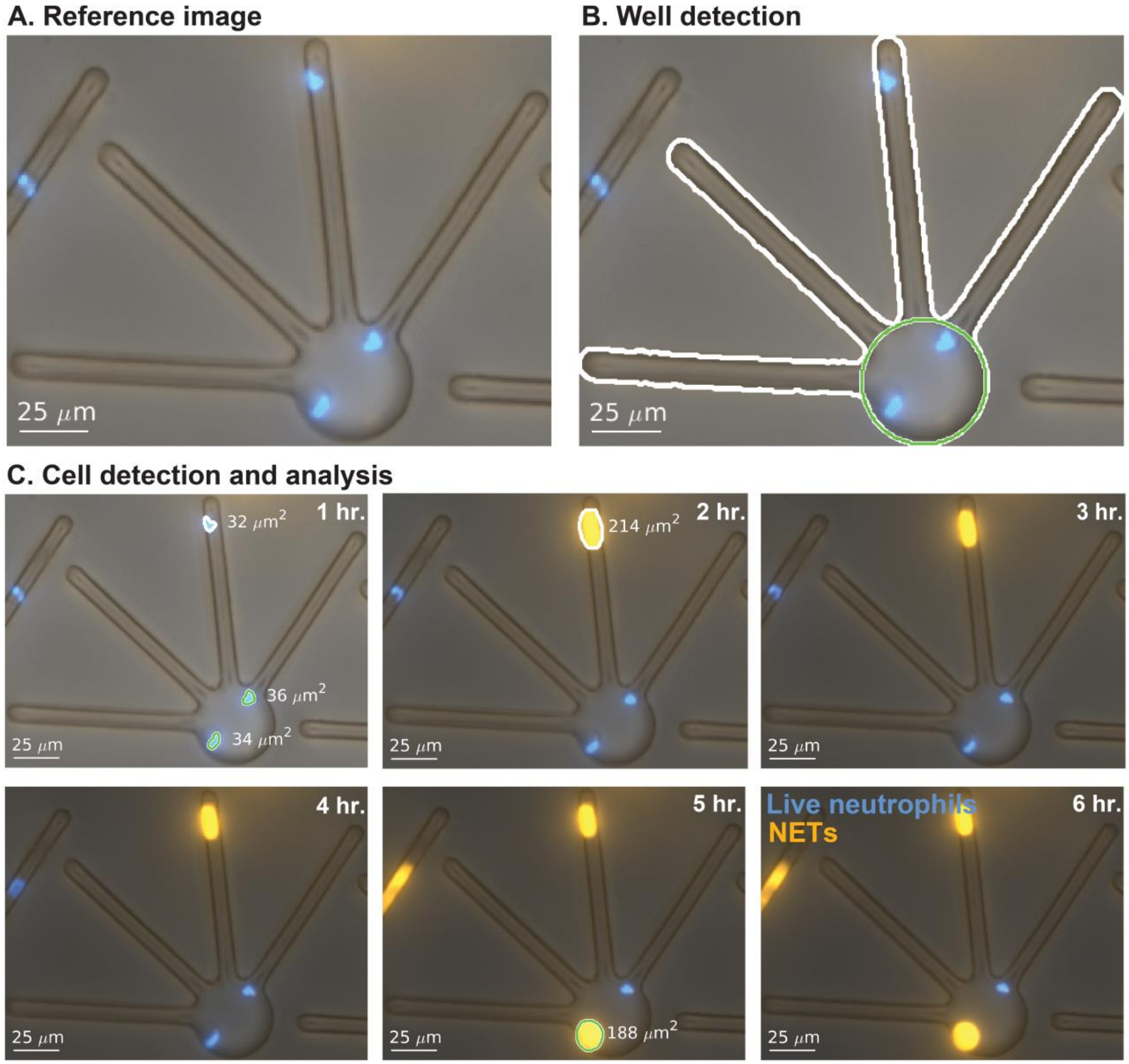

Figure S2. Computer-vision based image processing to automate the quantification of individual NETs released in different locations of the NET-array device. Blue: live neutrophil DNA (Hoechst 33342 stained); Orange: NETs (sytox orange stained). **(A)** Brightfield reference image of a single well overlayed with live cells at t = 1 hr. **(B)** Well detection followed by identification of chamber (boundary marked in green) and loop (boundary marked in white) **(C)** Cell detection of live (blue) and dead (orange) cells across all the time points. Area (in μm^2) of dead cells is computed at their first detection time point.

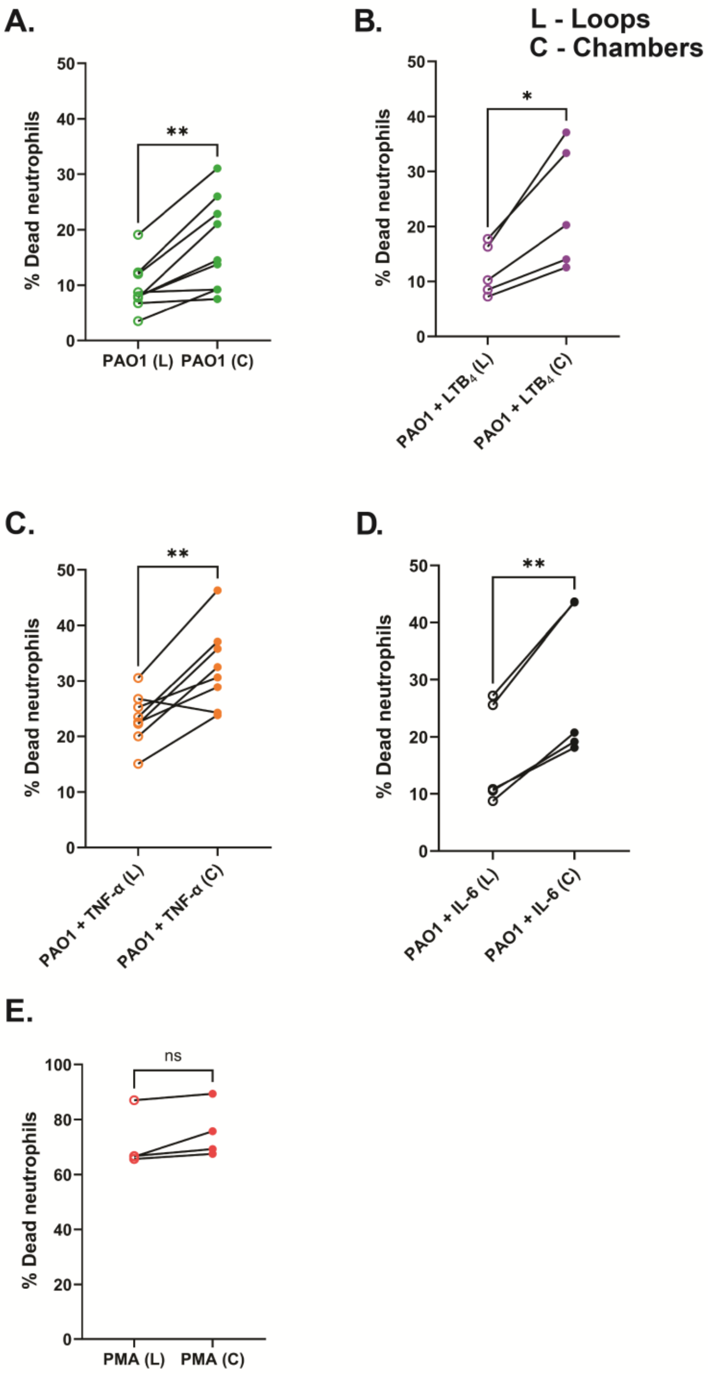

Figure S3. Comparative analysis showing lower percentage of dead neutrophils in the loops (L) relative to the chambers (C) of the device, when challenged with *P. aeruginosa* (PAO1) bacteria with or without the inflammatory mediators. (A) Neutrophils + PAO1 (no inflammatory mediator) **(B)** Neutrophils + PAO1 + 20 nM LTB_4 **(C)** Neutrophils + PAO1 + 20 ng/mL TNF-α and **(D)** Neutrophils + PAO1 + 50 ng/mL IL-6 and **(E)** Positive control (20 nM PMA). Data are represented as mean $\pm$ SEM and is collected from 5 to 9 separate healthy volunteers, except for positive control where n = 4 donors. ns – not significant, * $p < 0.05$, ** $p < 0.01$; two-tailed paired student t test used.

Video

Video S1. Video demonstrating the spatiotemporal dynamics of NETs released to *P. aeruginosa* (PAO1) bacteria in 5 × 5 wells of the NET-array device over a period of 6 hrs., under conditions listed. Blue: live neutrophil DNA (Hoechst 33342 stained); Orange: Dead cell DNA (sytox orange stained); Green: PAO1-GFP. **(A)** Negative control (neutrophils alone) **(B)** Positive control (20 nM PMA) **(C)** Neutrophils + PAO1 (no inflammatory mediator) **(D)** Neutrophils + PAO1 + 20 nM LTB_4 **(E)** Neutrophils + PAO1 + 20 ng/mL TNF-α and **(F)** Neutrophils + PAO1 + 50 ng/mL IL-6.
